# Machine Learning Algorithms to Predict Digital Competencies in University Faculty

**DOI:** 10.12688/f1000research.165342.1

**Published:** 2025-06-10

**Authors:** Jenniffer Sobeida Moreira-Choez, Aracelly Fernanda Núñez-Naranjo, Asia Cecilia Carrasco-Valenzuela, Héctor Luis López-López, Jesús Alejandro Vázquez Meza, Angel Ramón Sabando-García

**Affiliations:** 1 Facultad de Posgrado, Universidad Estatal de Milagro, Milagro, Guayas, 091050, Ecuador; 2 Centro de Investigación en Ciencias Humanas y de la Educación-CICHE. Facultad de Ciencias de la Educación, Universidad Tecnologica Indoamerica, Ambato, Tungurahua, 180103, Ecuador; 3 Universidad Autonoma de Sinaloa, Culiacán, Sinaloa, Mexico; 4 Departamento de Matemáticas y Estadística, Pontificia Universidad Catolica del Ecuador, Santo Domingo, Santo Domingo, Ecuador

**Keywords:** Machine learning, digital competence, higher education, artificial intelligence, teacher training, educational assessment, pedagogical innovation, educational technology.

## Abstract

**Background:**

The digital transformation of higher education has intensified the need to assess and enhance the digital competencies of university faculty. This study analyzed the effectiveness of various machine learning algorithms in predicting levels of faculty digital competence based on socio-educational variables. The objective was to develop an advanced predictive model, applied to faculty members from the State University of Milagro and the Technical University of Manabí.

**Methods:**

A quantitative approach was adopted, with a cross-sectional correlational design. Digital competencies were measured using the internationally validated DigCompEdu Check-In instrument, structured across six core dimensions. In the predictive phase, nine supervised machine learning algorithms were trained and evaluated: logistic regression, decision trees, random forest, gradient boosting, k-nearest neighbors, support vector machines, stochastic gradient descent, artificial neural networks, and Naive Bayes. The models were trained using a dataset comprising 4,154 observations, and their performance was assessed using standard classification metrics: area under the ROC curve (AUC), accuracy, F1-score, sensitivity, and Matthew’s correlation coefficient (MCC).

**Results:**

Gradient boosting, random forest, and neural network models demonstrated superior predictive performance, particularly at advanced competence levels (B2 and C1). Significant associations were identified between academic level, age, gender, and digital competencies. Logistic regression and Naive Bayes showed limitations in identifying low competence levels (A1), while intermediate levels were often overestimated across several models.

**Conclusions:**

The findings confirm that machine learning algorithms can accurately predict university faculty digital competencies. Advanced models outperformed traditional ones, especially at higher competence levels. It is recommended to incorporate contextual variables and validate the models in diverse educational settings.

## Introduction

The integration of digital technologies in higher education has led to a profound transformation in teaching methodologies and the development of teaching competencies ( Benavides et al., 2020; García-Morales et al., 2021; Moreira-Choez et al., 2024d). This shift responds not only to technological evolution but also to a pedagogical need to adapt to new educational paradigms that demand more effective and dynamic interaction within digital environments. Studies such as those by Moreira-Choez et al. (2024e) and Lindfors et al. (2021) emphasize how digitalization has reshaped expectations and traditional teaching methods, making continuous assessment of faculty digital competencies imperative. Furthermore, the variability in perception and competency levels among instructors, widely documented in the literatura ( Cattaneo et al., 2025; Tondeur et al., 2021), poses a significant challenge in terms of personalization and pedagogical effectiveness.

In this context, the application of machine learning algorithms offers transformative potential. The ability of these technologies to analyze large volumes of data and extract meaningful patterns can lead to accurate predictions about teachers’ digital competencies, thereby facilitating the design of more adaptive and effective professional training programs. According to Chen et al. (2020), the implementation of predictive models based on artificial intelligence has proven to enhance faculty adaptability to rapid and complex technological changes. Moreover, as Zhao et al. (2023) and Santamaria-Velasco et al. (2025), point out, these tools allow educational institutions to optimize resources and teaching strategies, ensuring better alignment with the needs and expectations of modern students. This technological and pedagogical convergence emerges as an essential pathway for advancing toward a higher education system that not only responds to technological imperatives but also promotes more inclusive and effective learning experiences ( Rane, 2025; Wei, 2023).

Nonetheless, evaluating digital competencies presents significant challenges ( Moreira-Choez et al., 2024c). Research by Garay-Rondero et al. (2024), highlights a noticeable deficiency in the availability of standardized tools to effectively assess these competencies across diverse academic disciplines. This lack of appropriate instruments hinders institutions’ ability to carry out accurate and consistent assessments of teachers’ digital skills. Additionally, current literature reveals a shortage of studies applying machine learning algorithms to effectively predict digital competencies in university settings, exposing a substantial gap in the existing body of research ( Essa et al., 2023; Hidalgo et al., 2020).

Given this scenario, the critical importance of developing predictive models based on machine learning algorithms becomes evident. This study seeks to address these research gaps through the application of advanced modeling techniques. It aims to deliver accurate and personalized predictions that foster a deeper understanding of university faculty’s digital competencies. Implementing such models can improve both the efficiency and effectiveness of competency assessments, while also providing valuable data for designing more effective pedagogical interventions and supporting continuous professional development. This methodological approach represents a significant step forward in adapting higher education to 21st-century demands, ensuring that educators are equipped with the necessary skills to navigate and thrive in an increasingly digitalized educational landscape.

To address the predictive capacity of machine learning algorithms on faculty digital competencies, the following research question is posed: ¿How can machine learning algorithms predict digital competencies among faculty at the State University of Milagro and the Technical University of Manabí? To answer this question, several guiding hypotheses are established:

H1. Machine learning algorithms can effectively predict university faculty’s digital competencies based on socio-educational variables such as age, gender, teaching experience, and academic level.

H2. There is a significant relationship between the academic level of university faculty and their digital competence, with higher levels observed among those with advanced academic qualifications.

H3. Machine learning models based on gradient boosting and neural networks are more accurate in predicting digital competencies compared to simpler models such as logistic regression and random forest.

H4. Differences in digital competencies among teachers are significantly influenced by demographic factors such as gender and age, with specific variations in their capacity to adapt to new technologies.

H5. Digital competencies related to assessment and feedback in digital learning environments are more difficult to predict using machine learning algorithms due to their complex and multifactorial nature.

To address the research question, this study proposes the development of an advanced machine learning model to predict digital competencies in faculty at the State University of Milagro and the Technical University of Manabí. The model aims not only to evaluate the effectiveness of various modeling techniques in predicting digital competencies but also to facilitate the implementation of more effective and personalized educational interventions, tailored to the specific needs and characteristics of the faculty. This research is framed as part of a broader effort to optimize teacher training and improve teaching and learning processes within increasingly digitalized educational contexts.

## Methods

This study adopted a quantitative approach with a cross-sectional and correlational design to investigate the effectiveness of machine learning algorithms in predicting digital competencies among university faculty at the State University of Milagro and the Technical University of Manabí. The relevance of this analysis lies in the growing imperative to effectively integrate digital technologies into teaching practices, which demands an accurate evaluation of faculty competencies in this domain. By implementing advanced modeling techniques, the study aimed to generate valuable insights to support professional development and promote continuous improvement in pedagogical practices.

To assess digital competencies, the DigCompEdu Check-In questionnaire was employed, adapted to the Spanish-speaking context by Cabero-Almenara and Palacios-Rodríguez (2020). This instrument, consisting of 22 items, evaluated six core areas of competence, ranging from professional engagement to the facilitation of students’ digital skills. Respondents’ scores were classified according to the DigCompEdu framework into proficiency levels from Newcomer (A1) to Pioneer (C2). The questionnaire was administered digitally via Google Forms and included an informed consent section that outlined the study’s objectives, ensuring voluntary participation and the confidentiality of the collected data. The informed consent process was conducted digitally and embedded within the same form as the questionnaire. Before responding to any items, participants encountered the informed consent statement at the beginning of the form. Only after providing explicit authorization an action restricted to individuals over 18 years of age were participants allowed to proceed with completing the questionnaire. In cases where consent was not granted, the form automatically concluded, thereby upholding ethical standards regarding informed and voluntary participation. The data collection process was conducted on February 28, 2025, when the instrument was applied to the study participants, following the established methodological and ethical procedures.

The machine learning phase involved the training of predictive models using a dataset comprising 4,154 observations, integrating data collected from both institutions. The training process incorporated nine supervised algorithms: logistic regression, decision trees, random forest, gradient boosting, k-nearest neighbors, support vector machines, stochastic gradient descent, artificial neural networks, and Naive Bayes. Model performance was evaluated through standard classification metrics, including area under the ROC curve (AUC), accuracy, F1-score, sensitivity, and Matthews correlation coefficient (MCC). This methodological design enabled the identification of significant patterns and associations, thereby reinforcing the empirical basis for institutional decision-making and faculty development planning.

**Figure 1.**
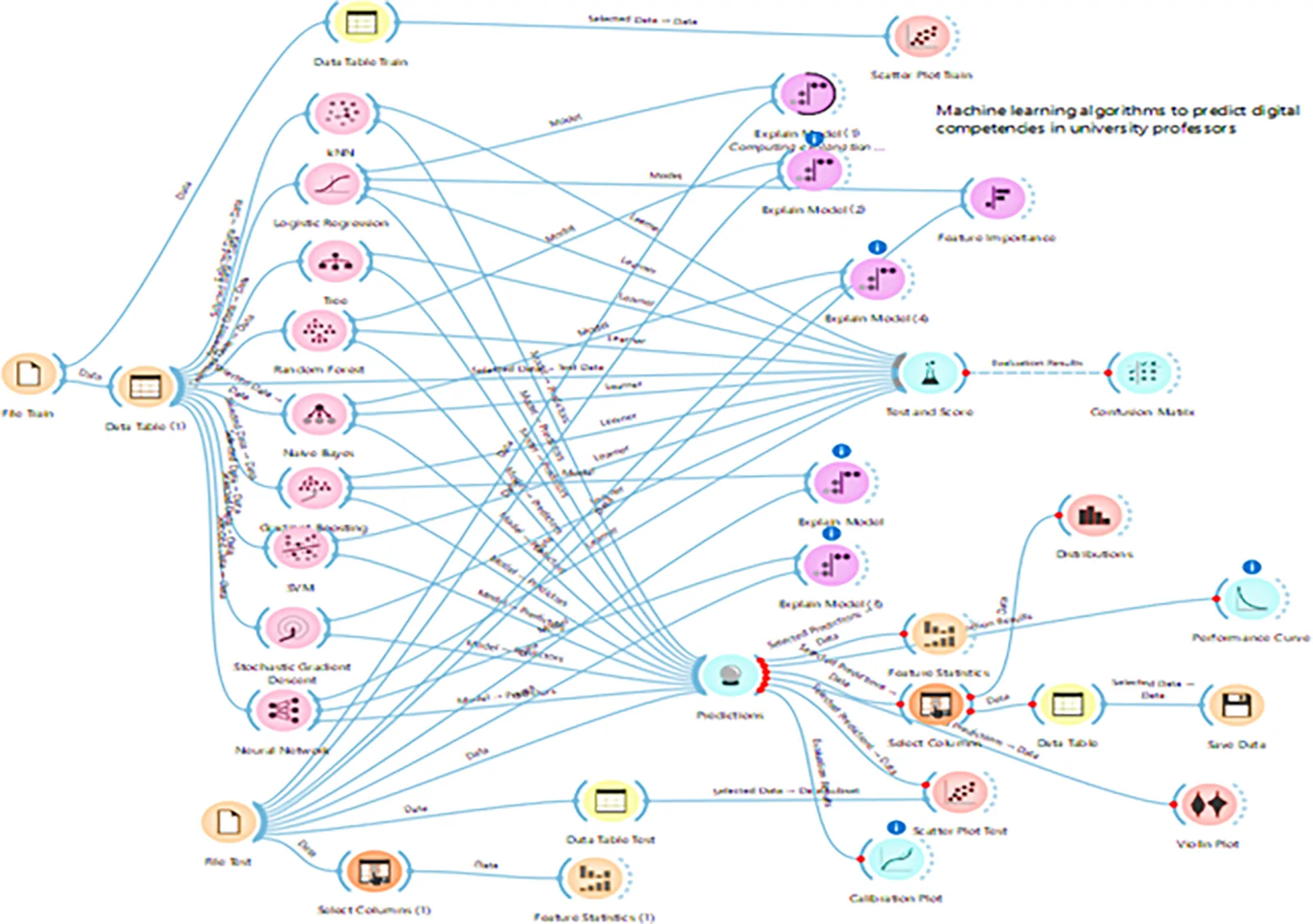
Process flow for predicting digital competencies using machine learning algorithms.

Figure 1 illustrates the methodology employed in the study on the use of machine learning algorithms to predict digital competencies among university faculty. The diagram outlines the analytical workflow from data input to model evaluation, emphasizing the integration of various modeling techniques for a comprehensive interpretation.

The Figure 1 illustrates the implementation of various machine learning models namely k-Nearest Neighbors (kNN), Logistic Regression, Random Forest, Naive Bayes, Gradient Boosting, Support Vector Machines (SVM), Stochastic Gradient Descent (SGD), and Neural Networks applied to predict digital competencies within an educational context. These models were developed and tested using the Orange Data Mining software (Orange version 3.38.1), an open-source platform designed for interactive data visualization and machine learning workflows. The visual programming interface provided by Orange enabled the sequential structuring of processes such as data preprocessing, model training, validation, and interpretation, facilitating reproducibility and transparency in the analytical procedures.

The predictive process commenced with a training phase, where structured datasets were introduced to calibrate the internal parameters of each algorithm. This was followed by a testing and evaluation phase, essential for determining each model’s predictive capacity in approximating real-world outcomes. To ensure reliability, models underwent rigorous validation protocols through the Test and Score and Confusion Matrix widgets. These components not only provided global accuracy metrics but also disaggregated performance indicators such as precision, recall, and F1-score, which are pivotal for assessing the models’ operational relevance in educational settings.

Furthermore, the Explain Model nodes within Orange enabled the dissection of feature contributions, offering interpretability by quantifying the influence of each predictor variable on the model’s output. This layer of analysis provided essential insights for educational researchers, allowing for a deeper understanding of the underlying structures driving predictive performance. The integration of explainability tools reinforced the analytical robustness and addressed ethical considerations regarding transparency in algorithmic decision-making.

Finally, the comparative analysis of models including logistic regression, random forest, gradient boosting, and neural networks revealed differential capacities in forecasting the digital competencies of university faculty. These algorithms were selected due to their scalability in managing high-dimensional data and their demonstrated efficacy in similar educational research contexts. The results contributed not only to the identification of the most performant models but also to the formulation of evidence-based recommendations for professional development programs, aiming to enhance digital proficiency among higher education instructors.

### Ethical considerations

In accordance with the ethical principles governing research involving human subjects, this study implemented a rigorous procedure to obtain informed consent, ensuring that all participants fully understood the nature, objectives, and implications of the study. It was clearly communicated that participation was entirely voluntary and that individuals were free to decline or withdraw from the research process at any time, without facing any negative consequences. To preserve confidentiality, personal data were anonymized, thus preventing any form of direct or indirect identification of the participants involved. Formal approval for this research was granted by the Institutional Review Board (IRB) of Milagro State University, as documented in the official resolution UNEMI-VICEINVYPOSG-DP-233-2025-OF, dated February 14, 2025.

To ensure scientific validity and the reproducibility of findings, the study was conducted in accordance with methodological guidelines that promote transparency throughout all phases of the research process. The adoption of these standards aims to strengthen the credibility of quantitative research by enabling the verification and replication of results by other scholars in similar contexts. Accordingly, standardized protocols were employed for both data collection and analysis, ensuring a rigorous and systematic approach free from subjective interference. This methodological strategy minimized potential biases and fostered an objective interpretation of the findings, in alignment with the principles of scientific integrity.

## Results and discussion

This section of the study presents findings related to the predictive capacity of machine learning algorithms in assessing the digital competencies of university faculty. The analysis explored how various socio-educational factors influence these competencies, using a dataset representative of the teaching population at the State University of Milagro and the Technical University of Manabí. Table 1 provides a detailed statistical analysis examining how different elements of digital competencies correlate with the academic level of university faculty. Competencies are categorized into key areas such as professional engagement, digital resources, digital pedagogy, assessment and feedback, and learner empowerment. The Chi-square (X ^2^) values and p-values provide evidence of the statistical significance of the observed relationships.

**Table 1.** Relationship between digital competencies and academic level of university faculty.

Competencies	Items	X ^2^	p
**Professional Engagement**	I systematically use different digital channels to improve communication with students, families, and colleagues. For example: email, messaging apps like WhatsApp, blogs, school websites.	19,213	0,004
	I use digital technologies to collaborate with my colleagues both within and outside my educational organization.	91,29	0,000
	I actively develop my own digital competence.	16,571	0,011
	I participate in online training courses. For example: government-provided online courses, MOOCs, webinars, etc.	51,435	0,000
**Digital Resources**	I use different websites and search strategies to find and select a wide range of digital resources.	27,048	0,000
	I create my own digital resources and adapt existing ones to meet my teaching needs.	66,851	0,000
	I securely protect sensitive content. For example: exams, grades, personal data.	58,143	0,000
**Digital Pedagogy**	I carefully consider how, when, and why to use digital technologies in class to ensure their added value is realized.	31,591	0,000
	I monitor student activities and interactions in the online collaboration environments we use.	50,295	0,000
	When my students work in groups or teams, they use digital technologies to acquire and document knowledge.	34,755	0,000
	I use digital technologies to enable students to plan, document, and evaluate their own learning. For example: self-assessment tests, digital portfolios, blogs, forums.	95,92	0,000
**Assessment and Feedback**	I use digital assessment strategies to monitor students' progress.	30,752	0,000
	I analyze all available data to identify students who need additional support. “Data” includes: student participation, performance, grades, attendance, activities, and social interactions in online environments. “Students who need additional support” refers to those at risk of dropout, low performance, learning disorders, specific learning needs, or lacking transversal skills (social, verbal, or study skills).	17,923	0,022
	I use digital technologies to provide effective feedback.	56,459	0,000
**Student Empowerment**	When proposing digital tasks, I consider and address potential issues such as equal access to devices and digital resources; compatibility problems or students’ low digital competence.	31,221	0,000
	I use digital technologies to offer students personalized learning opportunities. For example: assigning different digital tasks to address individual learning needs, taking into account preferences and interests.	45,048	0,000
	I use digital technologies to ensure active student participation in class.	45,53	0,000
**Facilitating Students’ Digital Competence**	I teach students how to evaluate the reliability of online information and to identify false and/or biased information.	49,383	0,000
	I propose tasks that require students to use digital media to communicate and collaborate with each other or with an external audience.	58,195	0,000
	I propose tasks that require students to create digital content. For example: videos, audio recordings, photos, presentations, blogs, wikis.	57,395	0,000
	I teach students how to behave safely and responsibly online.	34,752	0,000
	I encourage students to use digital technologies creatively to solve specific problems. For example, overcoming obstacles or emerging challenges in their learning process.	24,101	0,001

Note: Academic level (Undergraduate: 8.5%, Master's degree: 76.5%, Doctorate PhD: 15.0%). Competence level: (A1 Newcomer, A2 Explorer, B1 Integrator, B2 Expert, C1 Leader).

The results in the professional engagement category which include activities such as the use of various digital communication channels and participation in online training show statistically significant differences across academic levels, with particularly high Chi-square values. These findings are consistent with previous studies, such as those by Al-Rahmi et al. (2023), which indicate a correlation between academic level and the adoption of digital technologies in professional communication. Similarly, Maican et al. (2019) emphasize that faculty members with higher academic qualifications are more likely to employ advanced technologies for collaboration and communication, suggesting that research experience and training may influence both the ease and frequency with which new digital tools are adopted.

In the area of digital resources and digital pedagogy, the results reveal that activities such as creating personalized digital resources and supervising students in online collaborative environments vary significantly according to academic level. Bond et al. (2018) corroborate in their research that postgraduate-level instructors tend to integrate more complex technologies into their teaching methodologies, which is reflected in a greater predisposition to modify and adapt digital materials. Furthermore, Haleem et al. (2022) support the idea that higher academic levels are associated with more innovative pedagogical practices and a more strategic use of digital technologies to enhance learning processes.

In turn, digital assessment and feedback also show a strong association with academic level. Faculty members holding postgraduate and doctoral degrees use more sophisticated digital assessment strategies, aligning with the findings of Wang et al. (2021) who suggest that competence in digital assessment is greater among those with advanced academic training. This may be attributed to their greater exposure to learning environments that require and value precision in student performance evaluation and monitoring. Finally, the student empowerment category highlights how educators use digital technologies to personalize and enrich learning experiences. These results are supported by the research of Christodoulou and Angeli (2022), who found that faculty members with higher academic qualifications are more likely to employ digital technologies creatively to address pedagogical challenges, providing students with tools that foster autonomous and adaptive learning.

Table 2 presents a comprehensive analysis of the performance of various machine learning algorithms in predicting digital competencies, categorized by different competence levels ranging from A1 (Newcomer) to C1 (Leader). This breakdown facilitates an understanding of how each model performs in relation to the specific competence level of faculty members, offering critical insight into the effectiveness of each modeling technique.

**Table 2.** Evaluation of machine learning models by competence level of university faculty.

Model	AUC	CA	F1	Prec	Recall	MCC	Level
Logistic Regression	0.908	0.988	0.000	0.000	0.000	0.000	A1 (Newcomer)
Tree	0.920	0.988	0.000	0.000	0.000	0.000	
Gradient Boosting	0.920	0.988	0.000	0.000	0.000	0.000	
Random Forest	0.922	0.988	0.000	0.000	0.000	0.000	
kNN	0.518	0.988	0.000	0.000	0.000	0.000	
SVM	0.784	0.988	0.000	0.000	0.000	0.000	
SGD	0.500	0.988	0.000	0.000	0.000	0.000	
Neural Network	0.921	0.988	0.000	0.000	0.000	0.000	
Naive Bayes	0.693	0.988	0.000	0.000	0.000	0.000	
Logistic Regression	0.832	0.901	0.179	0.335	0.123	0.159	A2 (Explorer)
Tree	0.852	0.912	0.311	0.515	0.223	0.300	
Gradient Boosting	0.851	0.912	0.311	0.515	0.223	0.300	
Random Forest	0.852	0.912	0.311	0.515	0.223	0.300	
kNN	0.652	0.879	0.301	0.309	0.293	0.235	
SVM	0.588	0.555	0.186	0.111	0.573	0.072	
SGD	0.528	0.898	0.120	0.254	0.079	0.098	
Neural Network	0.852	0.912	0.311	0.515	0.223	0.300	
Naive Bayes	0.786	0.887	0.217	0.281	0.177	0.165	
Logistic Regression	0.669	0.692	0.141	0.224	0.103	-0.018	B1 (Integrator)
Tree	0.749	0.763	0.474	0.521	0.435	0.325	
Gradient Boosting	0.750	0.763	0.470	0.522	0.427	0.322	
Random Forest	0.750	0.763	0.470	0.522	0.427	0.322	
kNN	0.626	0.657	0.390	0.345	0.447	0.158	
SVM	0.599	0.661	0.240	0.267	0.219	0.025	
SGD	0.501	0.688	0.174	0.249	0.134	0.003	
Neural Network	0.749	0.763	0.474	0.521	0.435	0.325	
Naive Bayes	0.630	0.658	0.158	0.199	0.131	-0.047	
Logistic Regression	0.636	0.503	0.549	0.430	0.762	0.101	B2 (Expert)
Tree	0.691	0.584	0.584	0.485	0.735	0.220	
Gradient Boosting	0.683	0.584	0.580	0.484	0.722	0.213	
Random Forest	0.691	0.587	0.583	0.487	0.728	0.220	
kNN	0.557	0.555	0.474	0.447	0.504	0.091	
SVM	0.517	0.557	0.368	0.426	0.324	0.038	
SGD	0.556	0.532	0.534	0.442	0.675	0.113	
Neural Network	0.689	0.584	0.584	0.485	0.735	0.220	
Naive Bayes	0.620	0.558	0.543	0.461	0.660	0.149	
Logistic Regression	0.729	0.755	0.397	0.539	0.314	0.271	C1 (Leader)
Tree	0.759	0.775	0.451	0.601	0.361	0.337	
Gradient Boosting	0.758	0.770	0.458	0.579	0.379	0.332	
Random Forest	0.760	0.776	0.470	0.597	0.388	0.349	
kNN	0.621	0.721	0.313	0.424	0.248	0.161	
SVM	0.649	0.728	0.078	0.298	0.045	0.019	
SGD	0.620	0.721	0.431	0.452	0.411	0.247	
Neural Network	0.759	0.775	0.451	0.601	0.361	0.337	
Naive Bayes	0.719	0.736	0.438	0.482	0.402	0.269	

Data provided by respondents Moreira-Choez et al. (2025), processed with Orange version 3.38.1.

Table 2 reveals notable variability in model performance across different competence levels, with metrics such as AUC (Area Under the Curve), CA (Classification Accuracy), F1-score, Precision (Prec), Recall, and MCC (Matthews Correlation Coefficient). For instance, at the A1 (Newcomer) level, nearly all models except for Logistic Regression failed to accurately classify competencies, as evidenced by F1, Precision, and Recall values all being zero. This may suggest that the models are encountering difficulties in identifying distinguishing features at this initial level of competence, a finding consistent with studies by Smith and Zárate (1992) and Bansal et al. (2007), who observed that simple models often underperform in contexts where target categories are homogeneous or underrepresented.

As we move toward higher levels of competence, such as B2 (Expert) and C1 (Leader), some models particularly Random Forest and Gradient Boosting demonstrate substantial improvements in metrics like F1-score and Recall. This indicates that these models may be more suitable for contexts where inter-class differences are more pronounced and where a higher degree of discrimination is required, as noted by Asselman et al. (2023). Furthermore, the increase in MCC at higher levels suggests that these models are effective in balancing sensitivity and specificity, thereby providing more accurate and balanced predictions.

However, it is critical to note that, overall, models struggle to achieve high levels of accuracy at the lowest competence level (Newcomer), which may reflect a limitation in their ability to handle undifferentiated input data or features that are not clearly defined. This phenomenon underscores the importance of ongoing development and optimization of machine learning algorithms capable of effectively managing a broader range of data complexities ( Taye, 2023; Zhou et al., 2017). Moreover, advancing toward the incorporation of more sophisticated or hybrid modeling approaches is essential, particularly those capable of capturing nonlinear relationships and latent structures in educational datasets specially in segments where digital competencies are emerging or weakly differentiated. As Asselman et al. (2023) argue, the use of advanced models that integrate deep learning architectures or ensemble mechanisms can enhance the detection of subtle patterns, thereby enabling more accurate diagnostics and, consequently, the design of targeted, evidence-based pedagogical interventions. Such approaches also contribute to the development of adaptive systems that respond more effectively to the diversity of teaching profiles present within the higher education context.

**Figure 2.**
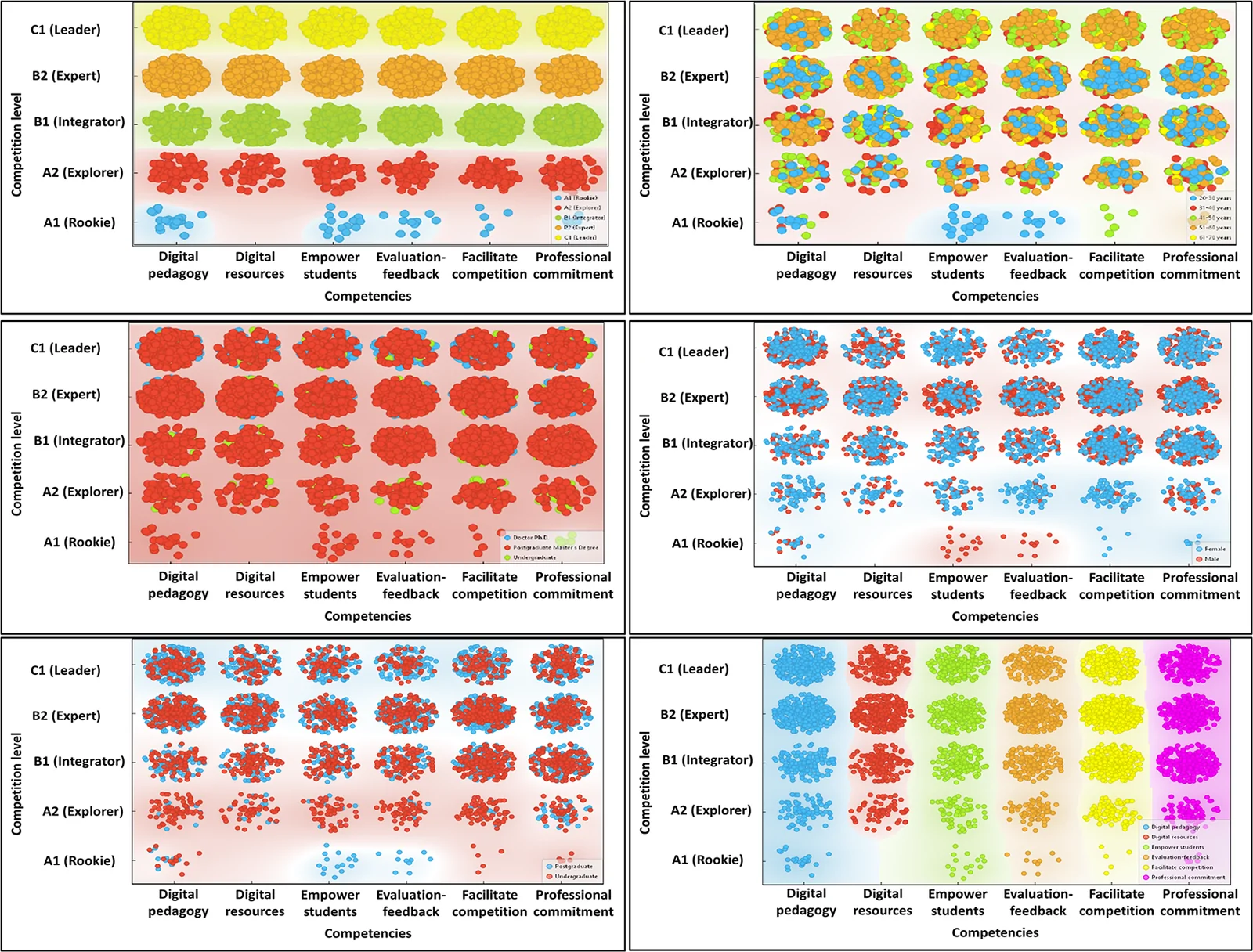
Competence level and type of competence according to socio-educational variables.

Figure 2 visually illustrates the correlation between teachers’ digital competence levels and various variables such as age, gender, teaching experience, and academic level. This graphical representation facilitates the understanding of trends and patterns emerging from the analyzed data.

Figure 2 presents a detailed visualization of university faculty members’ digital competence levels based on various socio-educational variables, allowing for the identification of relevant patterns in the distribution of digital skills. A significant concentration is observed at intermediate and advanced levels (B1, B2, and C1), particularly in competencies related to digital pedagogy, the use of digital resources, and feedback. This trend suggests that a considerable portion of faculty has developed key competencies necessary for the integration of technology into the teaching-learning process. However, substantial gaps persist at the basic levels (A1 and A2), indicating that segments of the academic population still require intensive support to reach satisfactory levels. This phenomenon is well-documented in the literature, where it is noted that digital inequalities continue to be reproduced, especially in institutions with pedagogical cultures centered on traditional methodologies ( Schmidt & Tang, 2020).

The distribution by age reveals that younger faculty members tend to be positioned at higher levels of digital competence, which may be associated with greater exposure to and familiarity with technological tools during their initial training. These findings reinforce those of Alcaide-Pulido et al. (2025), who identified a positive correlation between age and technological adaptability. Regarding gender, the figure shows slight disparities that may reflect structural inequalities in access to technological training opportunities, as highlighted in previous studies on gender gaps in digital competencies ( Stoet & Geary, 2018). Such differences underscore the need to incorporate an equity perspective into the design of professional development policies.

Academic level also emerges as a determining factor in the development of digital competencies. Faculty with postgraduate education, particularly those holding a doctoral degree, show a higher concentration at the B2 and C1 levels compared to those with only undergraduate training. This finding aligns with the results of Mei et al. (2019), who emphasized that higher academic qualifications are associated with a greater ability to critically and effectively incorporate digital technologies in educational settings. Thus, academic trajectory constitutes a key predictor of technological proficiency in teaching practice.

In light of these results, the need to design institutional strategies for continuous professional development is reinforced strategies that take into account both the initial level of competence and the socio-demographic characteristics of faculty members. It is not enough to promote the acquisition of technological tools; rather, a methodological reconfiguration is required to support a transition toward pedagogical approaches based on innovation and flexibility. In this regard, Rofi’i et al. (2023) argue that training programs must be adaptive and context-sensitive, while Vindigni (2023) emphasizes the importance of incorporating inclusive approaches that respond to educators’ personal and professional trajectories.

**Figure 3.**
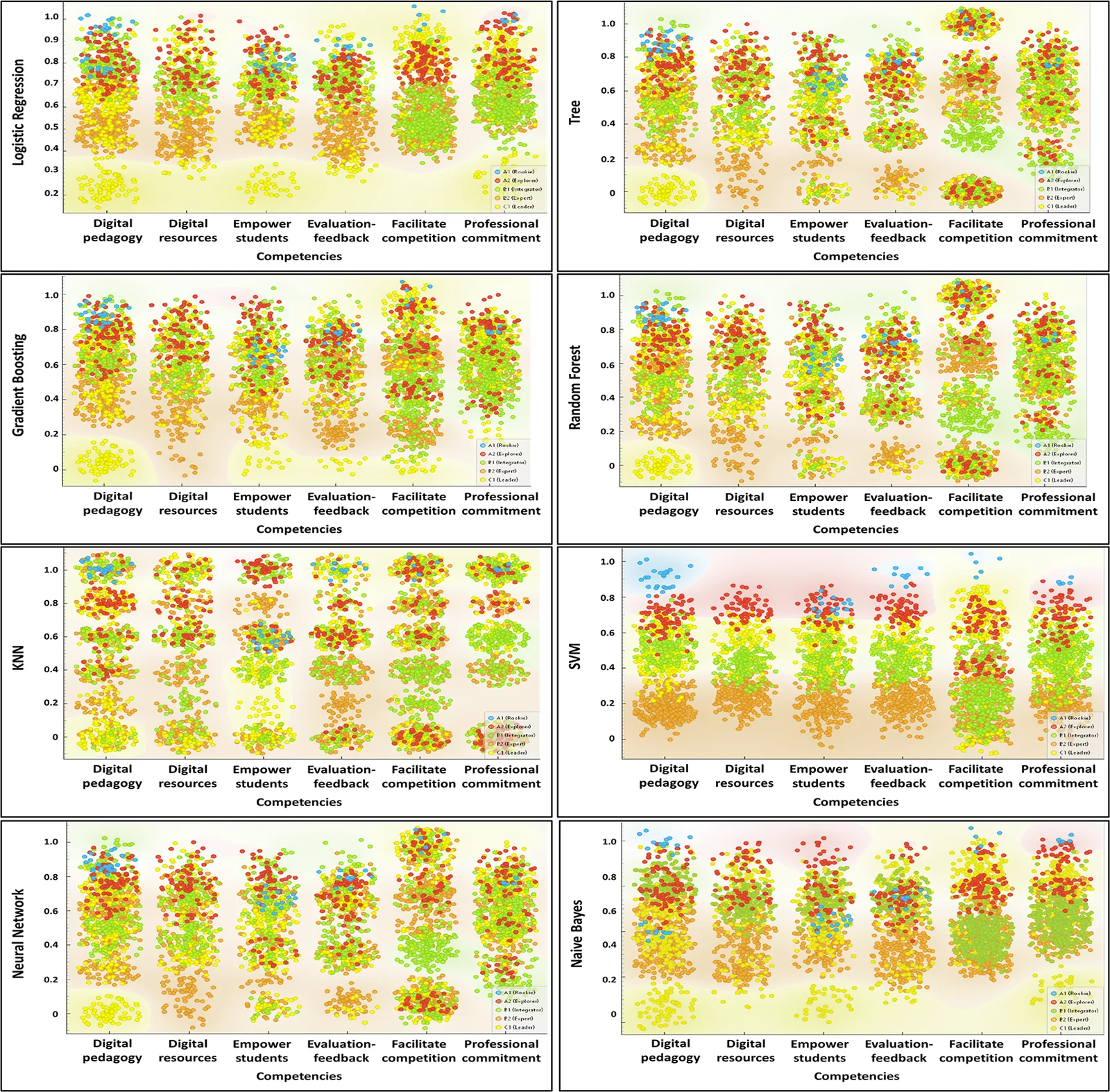
Performance of predictive models according to competence level and type of competence.

Figure 3 provides an in-depth visualization of how different machine learning models classify various digital competencies across different competence levels. Each panel within the figure represents a detailed comparison between types of competence and their distribution according to the model used, highlighting both accuracy and areas for improvement in the prediction of teachers’ specific digital skills.

Figure 3 provides a comparative view of the performance of various machine learning algorithms in predicting digital competencies, allowing for the observation of distinct patterns based on the type of competence evaluated. Overall, a higher density of accurate predictions is observed at advanced competence levels (B2 and C1), particularly in categories related to digital pedagogy, digital resources, and feedback, when using models such as Random Forest, Gradient Boosting, and Neural Networks. This trend can be attributed to the robustness of these algorithms in handling nonlinear relationships and complex data structures, which aligns with the findings of Dong et al. (2021), who argue that such models achieve better generalization when there is clear differentiation among latent classes.

In contrast, lower accuracy is observed at the more basic levels (A1 and A2), especially in models such as k-Nearest Neighbors (kNN) and Stochastic Gradient Descent (SGD), where predictions tend to be more dispersed and less aligned with actual values. This variability may be due to these models’ sensitivity to high-dimensional datasets or class imbalance, as noted by Kumar et al. (2023). Furthermore, the irregular performance of kNN in competencies such as student empowerment or facilitation of digital competence suggests that its effectiveness diminishes when categories have conceptual overlaps or lack well-defined structures. This observation highlights the importance of aligning the model with the specific type of competence being evaluated, recognizing that not all machine learning approaches yield consistent performance across all competency domains.

It is worth emphasizing that ensemble-based models, such as Random Forest and Gradient Boosting, not only offer higher predictive power but also provide greater stability across the various assessment areas of the DigCompEdu questionnaire. Their consistent performance across all dimensions indicates a superior ability to capture complex patterns and interactions among variables, which is crucial in diverse educational contexts. This characteristic is especially relevant for educational interventions, as it enables more accurate diagnostics and, therefore, the design of improvement strategies tailored to the specific digital profile of the instructor. Alghamdi et al. (2025) argue that the practical utility of predictive models lies in their ability to precisely identify individual weaknesses, thereby facilitating the implementation of more targeted and effective training plans.

**Figure 4.**
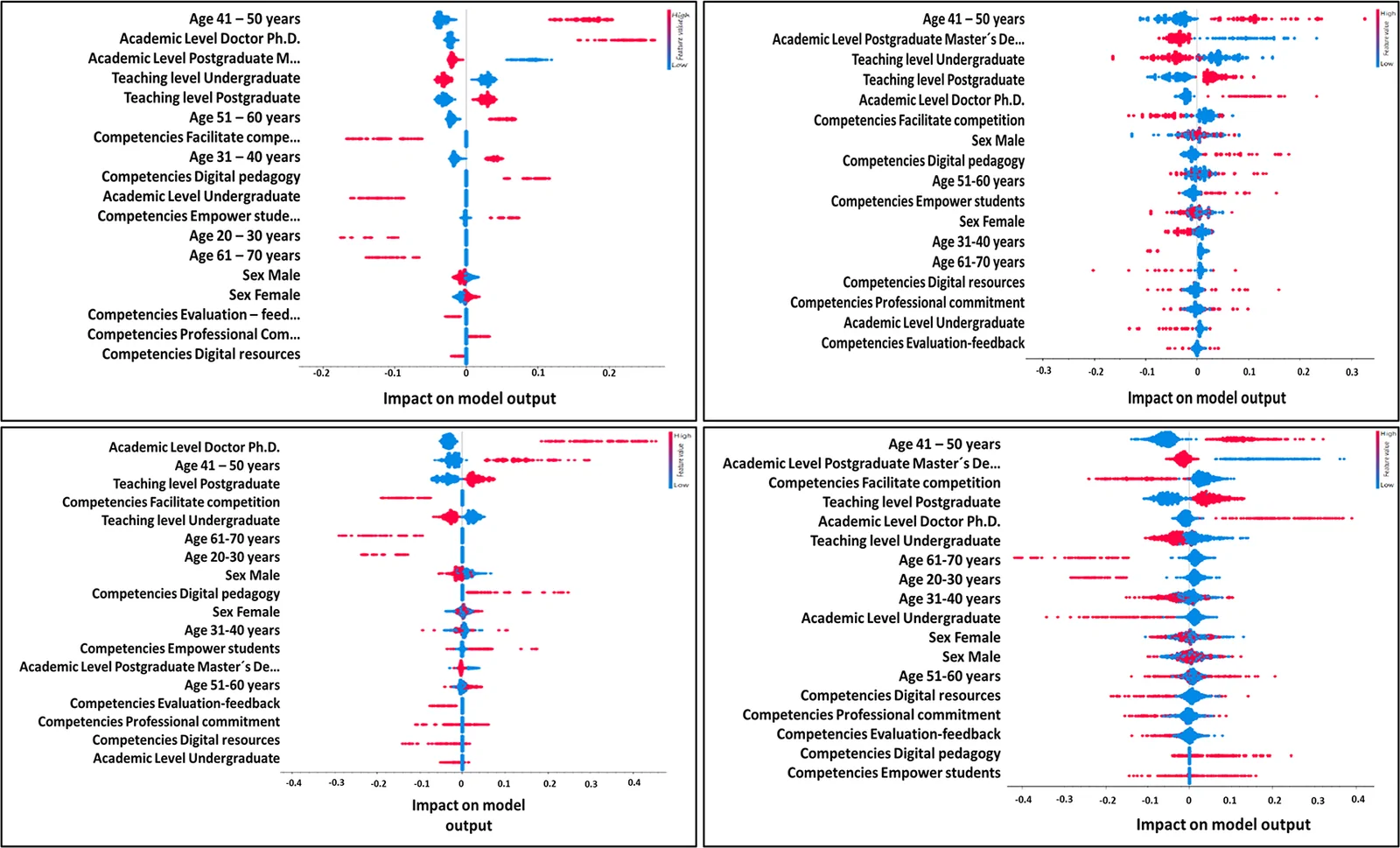
Comparative analysis of the impact of socio-educational variables on four machine learning models.

Figure 4 presents a comparative analysis of the impact of various socio-educational variables on the performance of four different machine learning models, enabling a detailed assessment of how factors such as age, gender, and academic level influence digital competence predictions in educational settings. This comparison highlights the variability in the influence of these variables across models, underscoring the inherent complexity of modeling digital competencies in the educational domain.

Figure 4 shows that middle-aged and older age groups specifically those between 41–50 and 51–60 years as well as higher academic levels such as Ph.D. and Master’s degree holders, have a positive impact on most of the models analyzed. These findings align with current literature, such as the study by Thordsen and Bick (2023), which highlights the correlation between academic maturity and greater technological integration. This pattern suggests that professional experience and prolonged development may facilitate more efficient and strategic adoption of technological tools in teaching.

On the other hand, the analysis reveals significant differences in the impact of gender variables across the models, reflecting potential variations in technology access and usage between men and women in educational environments. Studies by Choudhary (2024) support this observation, arguing that training programs should be tailored to address these differences, ensuring that digital competence interventions are inclusive and effective. Moreover, the results indicate a negative impact of the variables 20–30 years and Undergraduate in several model configurations, suggesting a deficiency in technological training at the early stages of academic careers. This challenge is recognized in the research of Chohan and Hu (2022), who propose strengthening digital education from initial training levels to mitigate competence gaps from the outset.

**Figure 5.**
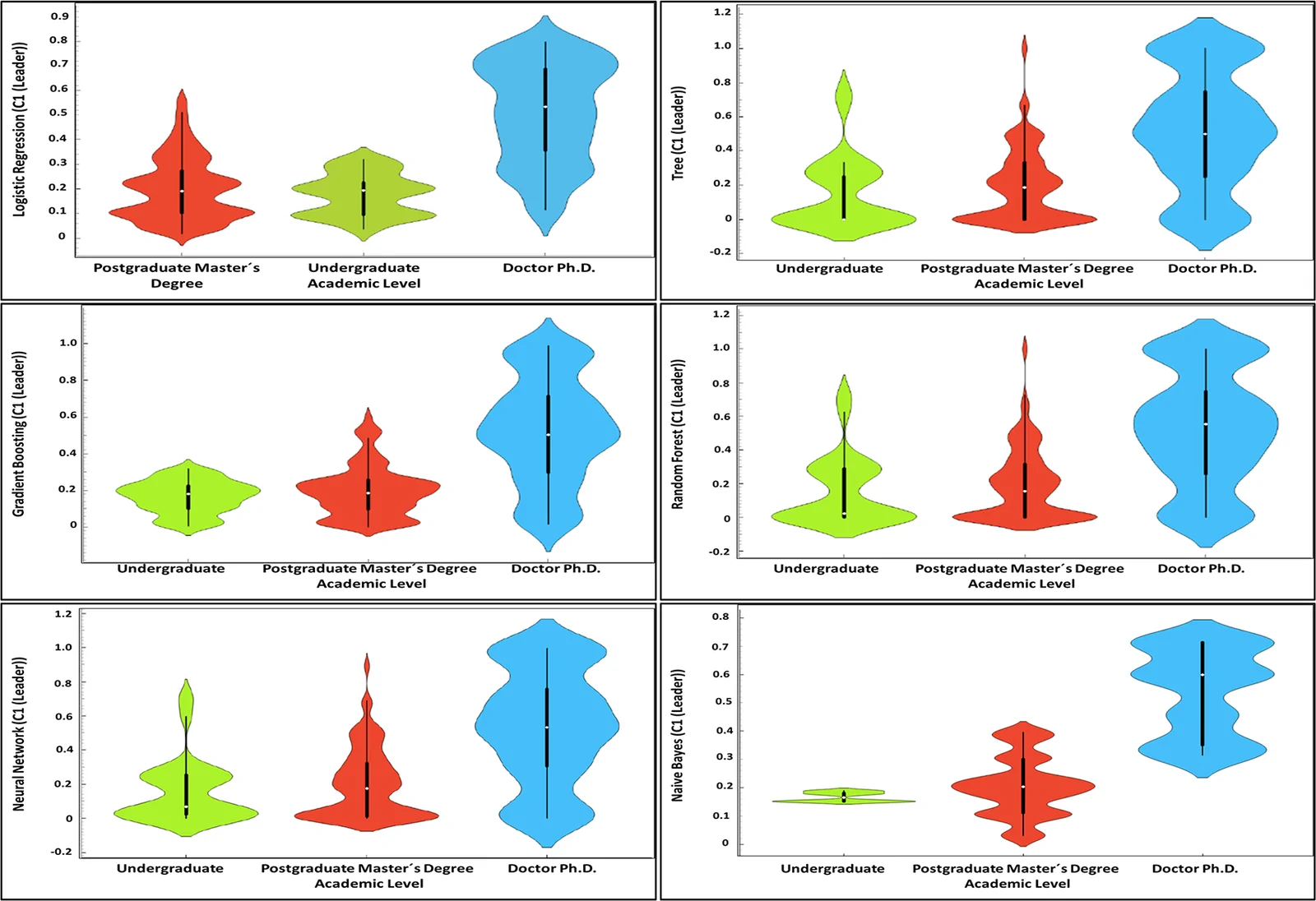
Distribution of leader-level competencies according to academic level.

Figure 5 provides a visual analysis using violin plots that illustrate the distribution of advanced (leader-level) digital competencies based on the academic level of university faculty. This visualization enables a comparison of the density and dispersion of competencies across three distinct academic categories: Undergraduate, Master’s Degree, and Ph.D.

The violin plots illustrate notable differences in the distribution of leader-level competencies across academic levels. Faculty members holding Ph.D. degrees display a wider and more symmetrical distribution, indicating greater uniformity and breadth in their digital competencies. This finding supports the research by Palacios-Rodríguez et al. (2024), which suggests that instructors with higher educational qualifications tend to possess more developed digital skills due to increased exposure to advanced technologies and research methodologies during their doctoral training.

In contrast, the plots for Undergraduate and Master’s Degree levels show narrower and more asymmetrical distributions, which may indicate variability in digital competence levels within these groups. This could reflect less consistency in the integration of digital technologies into undergraduate and graduate curricula, a point emphasized by Tee et al. (2024), who argue that disparities in technological training during the early years of higher education can lead to significant gaps in digital competencies. Additionally, the comparison of the tails of the plots shows that, although some individuals at the Undergraduate and Master’s levels achieve competence levels comparable to Ph.D. holders, the majority are concentrated in lower ranges. This pattern is consistent with findings by Radovan and Radovan (2024), who observed that opportunities for ongoing professional development and access to technological resources are less frequent at these educational levels, potentially limiting the development of advanced competencies.

**Figure 6.**
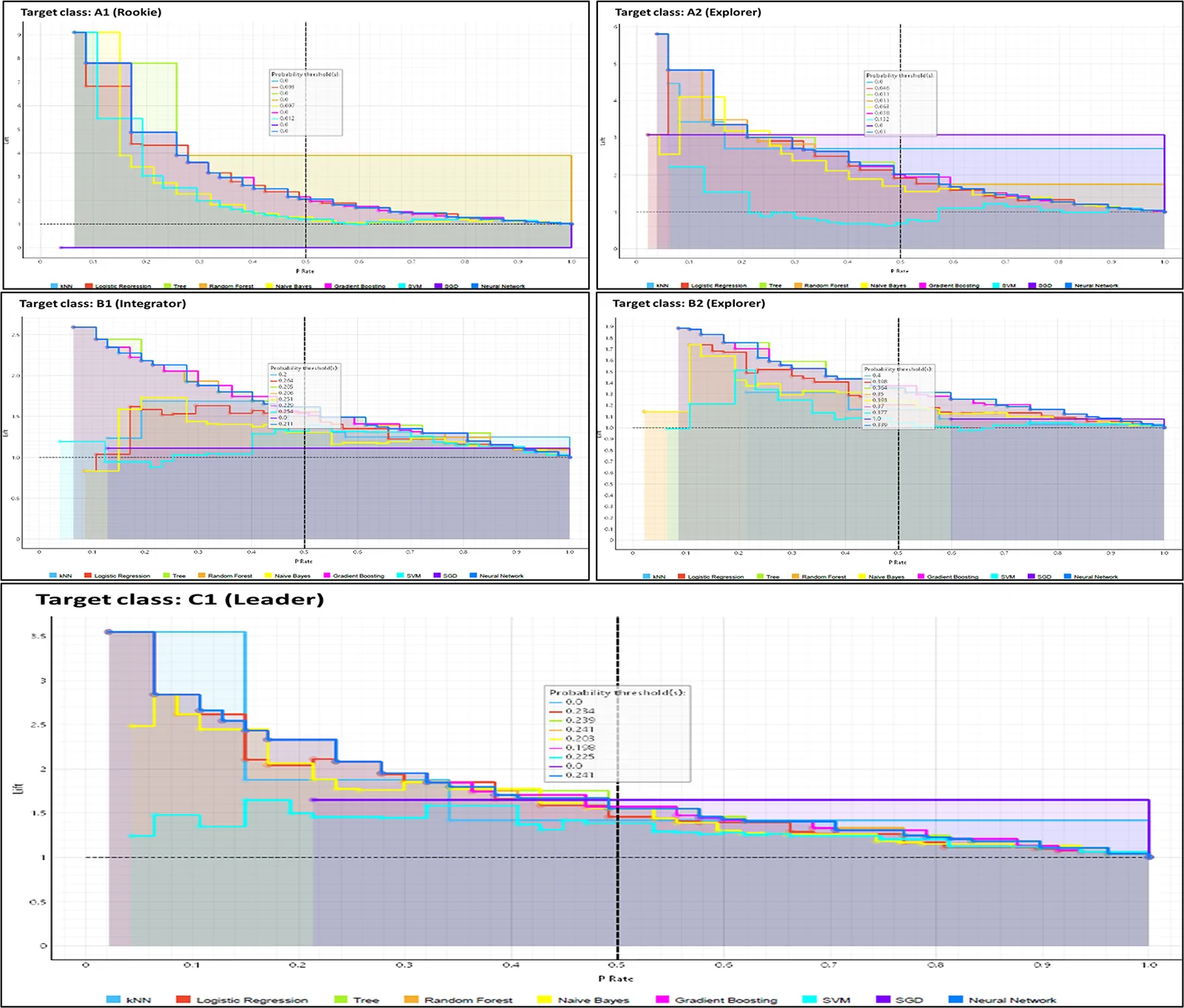
Calibration of models at the levels of digital competencies.

Figure 6 presents a detailed comparison of the calibration of various machine learning models, including logistic regression, decision trees, and gradient boosting, among others. This visual analysis demonstrates how each model predicts digital competencies and evaluates the accuracy of these predictions by aligning them with actual observations.

Neural network and gradient boosting models, which are closer to the diagonal line in the calibration plots, indicate greater accuracy in probability estimation. This result suggests that such models are better suited to handling the inherent complexities of educational data. The effectiveness of these advanced models in predicting digital competencies is supported by studies such as Fakhar et al. (2024), who highlight their superior capacity to adapt to complex patterns and interdependent variables in educational settings. This perspective is further reinforced by Alnasyan et al. (2024), who argue that deep learning techniques, such as neural networks, are particularly effective in capturing nonlinear and multifaceted dynamics that often characterize educational data. Moreover, the study by Beer and Mulder (2020) illustrates how the application of gradient boosting has improved the prediction of educational outcomes by fitting models that consider a wide range of influential factors, thus demonstrating the importance of using sophisticated methods in educational assessment.

In contrast, simpler models notably Logistic Regression, Decision Trees, and k-Nearest Neighbors (kNN) display significant divergence from the diagonal. This misalignment reflects underfitting, where the models fail to capture the underlying structures in the data. As emphasized by Islam et al. (2025), traditional models often lack the flexibility required to adapt to the multifaceted nature of educational data, particularly when the classification task involves nuanced levels of digital proficiency. Moreover, Awedh and Mueen (2025) point out that such models are generally ineffective at identifying latent patterns or dealing with overlapping class boundaries, thereby reducing their predictive power and practical utility in educational assessment.

Table 3 presents a detailed comparison of the main machine learning algorithms used to predict levels of digital competence among faculty. Each confusion matrix displays the proportions of classifications made by the models in relation to the actual and predicted competence levels, ranging from A1 (Newcomer) to C1 (Leader). The analysis includes models such as logistic regression, decision trees, gradient boosting, random forest, k-nearest neighbors (kNN), support vector machines (SVM), stochastic gradient descent (SGD), neural networks, and naive Bayes.

**Table 3.** Comparative confusion matrix of predictive models for faculty digital competence levels.

Actual Level	A1 (Newcomer)	A2 (Explorer)	B1 (Integrator)	B2 (Expert)	C1 (Leader)	Σ	Actual Level	A1 (Newcomer)	A2 (Explorer)	B1 (Integrator)	B2 (Expert)	C1 (Leader)	Σ
**Logistic Regression Model**							**Decision Tree Model**						
A1 (Newcomer)	NA	2.4%	1.9%	1.2%	0.0%	60	A1 (Newcomer)	NA	0.0%	0.6%	1.8%	0.0%	60
A2 (Explorer)	NA	33.5%	28.6%	6.5%	0.0%	457	A2 (Explorer)	NA	51.5%	7.2%	9.0%	0.0%	457
B1 (Integrator)	NA	37.7%	22.4%	27.7%	8.4%	1,263	B1 (Integrator)	NA	29.3%	52.1%	18.8%	9.1%	1,263
B2 (Expert)	NA	17.4%	29.1%	43.0%	37.7%	2,048	B2 (Expert)	NA	19.2%	24.7%	48.5%	30.8%	2,048
C1 (Leader)	NA	9.0%	18.1%	21.6%	53.9%	1,320	C1 (Leader)	NA	0.0%	15.5%	21.9%	60.1%	1,320
Σ	0	167	581	3,630	770	5,148	Σ	0	198	1,056	3,102	792	5,1
**Decision Tree Model**							**Random Forest Model**						
A1 (Newcomer)	NA	0.0%	0.5%	1.8%	0.0%	60	A1 (Newcomer)	NA	0.0%	0.5%	1.8%	0.0%	60
A2 (Explorer)	NA	51.5%	7.4%	9.1%	0.0%	457	A2 (Explorer)	NA	51.5%	7.4%	9.1%	0.0%	457
B1 (Integrator)	NA	29.3%	52.2%	19.1%	9.5%	1,263	B1 (Integrator)	NA	29.3%	52.2%	19.3%	8.8%	1,263
B2 (Expert)	NA	19.2%	24.2%	48.4%	32.6%	2,048	B2 (Expert)	NA	19.2%	24.2%	48.7%	31.5%	2,048
C1 (Leader)	NA	0.0%	15.8%	21.5%	57.9%	1,320	C1 (Leader)	NA	0.0%	15.8%	21.1%	59.7%	1,320
Σ	0	198	1,033	3,054	863	5,148	Σ	0	198	1,033	3,060	857	5,148
**k-Nearest Neighbors (kNN) Model**							**SVM Model**						
A1 (Newcomer)	NA	0.2%	1.4%	1.3%	0.8%	60	A1 (Newcomer)	NA	1.2%	0.6%	1.7%	0.0%	60
A2 (Explorer)	NA	30.9%	11.1%	3.9%	6.6%	457	A2 (Explorer)	NA	11.1%	8.8%	6.7%	0.9%	457
B1 (Integrator)	NA	27.7%	34.5%	20.3%	14.1%	1,263	B1 (Integrator)	NA	27.1%	26.7%	20.3%	16.7%	1,263
B2 (Expert)	NA	31.6%	36.7%	44.7%	36.1%	2,048	B2 (Expert)	NA	39.2%	34.2%	42.6%	53.5%	2,048
C1 (Leader)	NA	9.5%	16.2%	29.7%	42.4%	1,320	C1 (Leader)	NA	21.4%	29.8%	28.9%	29.8%	1,320
Σ	0	198	1,033	3,054	863	5,148	Σ	0	2,357	1,035	1,558	198	5,148
**SGD Model**							**Neural Network Model**						
A1 (Newcomer)	NA	2.1%	2.5%	1.1%	0.6%	60	A1 (Newcomer)	NA	0.0%	0.5%	1.8%	0.0%	60
A2 (Explorer)	NA	25.4%	24.9%	6.5%	4.1%	457	A2 (Explorer)	NA	51.5%	7.2%	9.0%	0.0%	457
B1 (Integrator)	NA	22.5%	24.9%	27.9%	15.7%	1,263	B1 (Integrator)	NA	29.3%	52.1%	18.9%	9.1%	1,263
B2 (Expert)	NA	28.9%	31.2%	44.2%	34.4%	2,048	B2 (Expert)	NA	19.2%	24.7%	48.5%	30.8%	2,048
C1 (Leader)	NA	21.1%	16.6%	20.3%	45.2%	1,320	C1 (Leader)	NA	0.0%	15.5%	21.9%	60.1%	1,320
Σ	0	142	680	3,128	1,198	5,148	Σ	0	198	1,056	3,102	792	5,148

Actual Level	A1 (Newcomer)	A2 (Explorer)	B1 (Integrator)	B2 (Expert)	C1 (Leader)	Σ
**Naive Bayes Model**						
A1 (Newcomer)	NA	1.7%	2.1%	1.3%	0.0%	60
A2 (Explorer)	NA	28.1%	25.6%	5.6%	0.0%	457
B1 (Integrator)	NA	40.6%	19.9%	27.0%	17.3%	1,263
B2 (Expert)	NA	20.1%	31.2%	46.1%	34.5%	2,048
C1 (Leader)	NA	9.4%	21.3%	20.0%	48.2%	1,320
Σ	0	288	828	2,932	1,100	5,148

The results in Table 3 show that the Gradient Boosting, Random Forest, and Neural Network models demonstrated a higher ability to correctly predict the upper levels of digital competence (B2 and C1), particularly in the case of the Gradient Boosting model, which achieved a correct classification rate of 57.9% for instructors at the C1 (Leader) level. This performance aligns with previous findings highlighting the effectiveness of these models in handling nonlinear relationships and complex data structures ( Kukkar et al., 2019; Kyriazos & Poga, 2024). In contrast, the Naive Bayes model showed clear limitations, particularly at the lower levels, systematically underestimating actual competence levels. This is consistent with studies indicating its limited capacity to capture interactions among predictive variables in complex educational contexts ( Almalawi et al., 2024; Sharma et al., 2019).

A general trend of overprediction at intermediate levels (B1 and B2) was also observed across most algorithms, suggesting ambiguity in classifying instructors with mid-range competence profiles. This phenomenon has been reported by Moreira-Choez et al. (2024b), who note that intermediate levels of digital competence tend to exhibit greater variability and dispersion based on factors such as age, gender, and teaching experience. In this regard, the robustness of more advanced models allowed for better capture of transitions between levels and more accurate differentiation of instructors at the threshold of digital competence advancement.

Ultimately, these findings reinforce the importance of employing high-complexity models for predictive diagnostic tasks in educational environments where multidimensional constructs such as digital competencies are being analyzed. The comparison of confusion matrices clearly demonstrates that models like Gradient Boosting and Random Forest not only offer higher overall accuracy but also reduce misclassification errors between adjacent levels. This contributes to more effective planning of personalized training interventions ( Ingkavara et al., 2022; Moreira-Choez et al., 2024a). These results provide valuable empirical evidence to support future applications of artificial intelligence in faculty assessment throughout Latin America.

## Conclusions

The present study fulfilled its objective of developing and evaluating machine learning models aimed at predicting digital competencies among university faculty, based on socio-educational variables. Furthermore, the research question was addressed by demonstrating that the implemented algorithms particularly Gradient Boosting, Random Forest, and Neural Networks achieved superior performance in terms of accuracy, especially when classifying intermediate and advanced levels of digital competence. These findings provide a solid empirical foundation for the application of artificial intelligence in assessing professional development in higher education contexts.

Regarding the hypotheses proposed, the results supported their validation in most cases. It was confirmed that machine learning algorithms can effectively predict faculty digital competencies based on variables such as age, gender, teaching experience, and academic degree. A significant relationship between academic level and digital competence was verified, with higher competencies observed among faculty with postgraduate or doctoral training. Advanced models, particularly Gradient Boosting and Neural Networks, proved to be more accurate compared to simpler models such as Logistic Regression and Naive Bayes. Additionally, it was found that digital competencies are influenced by demographic factors, with notable differences by age and gender. It was also corroborated that competencies related to digital assessment and feedback were more difficult to predict, suggesting a multifactorial nature requiring deeper analytical approaches.

Among the most relevant findings, it was identified that the Gradient Boosting model achieved the highest levels of accuracy in correctly classifying faculty at the C1 (Leader) and B2 (Expert) competence levels. However, all models showed limitations in classifying the A1 (Novice) level, highlighting a lower sensitivity to profiles with emerging digital skills. This trend reinforces the need to refine model performance at the extremes of the competence scale, where data distribution tends to be scarcer and more heterogeneous.

The study presented certain limitations, particularly regarding sample composition, such as the underrepresentation of instructors at lower competence levels and the absence of contextual variables that could enrich the analysis. This situation affected the generalizability of results in specific segments and limited the interpretation of institutional or disciplinary factors influencing digital competencies.

As a future direction, it is recommended to implement hybrid models integrating both supervised and unsupervised techniques, as well as to explore deep learning algorithms to improve the sensitivity of predictions across all levels. Furthermore, it is suggested to extend the empirical validation of these models across diverse educational contexts, considering both pedagogical and organizational variables. This line of research will contribute to the development of more precise analytical tools for the diagnosis and enhancement of digital teaching competencies in Latin America.

## Data Availability

Figshare: Data from the article titled Machine Learning Algorithms to Predict Digital Competencies in University Faculty. https://doi.org/10.6084/m9.figshare.29036066.v1 ( Moreira Choez et al., 2025). The project contains the following underlying data:
- Data from the article titled Machine Learning Algorithms to Predict Digital Competencies in University Faculty.xlsx Data are available under the terms of the Creative Commons Zero “No rights reserved” data waiver (CC0 1.0 Public domain dedication).
