# Supplementary material for: Machine learning algorithms to predict digital skills in university teaching staff
Source: F1000Res. 2026 Jul 11;14:573. Originally published 2025 Jun 10. [Version 2] doi: 10.12688/f1000research.165342.2 (PMC13560009; doi:10.12688/f1000research.165342.2)
Supplement: Peer review history

# Reviewer report: Reviewer response for version 2

**DOI:** 10.5256/f1000research.203365.r500524

**Published:** 2026-08-14

**Authors:** William J Marin-Rodriguez

**Affiliations:** 1 Universidad Nacional José Faustino Sánchez Carrión, Huacho, Peru

Approved

Is the work clearly and accurately presented and does it cite the current literature?

Yes

If applicable, is the statistical analysis and its interpretation appropriate?

Partly

Are all the source data underlying the results available to ensure full reproducibility?

Yes

Is the study design appropriate and is the work technically sound?

Partly

Are the conclusions drawn adequately supported by the results?

Partly

Are sufficient details of methods and analysis provided to allow replication by others?

Partly

Reviewer Expertise:

Data science

I confirm that I have read this submission and believe that I have an appropriate level of expertise to confirm that it is of an acceptable scientific standard.

# Reviewer report: Reviewer response for version 2

**DOI:** 10.5256/f1000research.203365.r500523

**Published:** 2026-07-13

**Authors:** Giorgia Montanucci, Talia Sbardella

**Affiliations:** 1 University for Foreigners of Perugia, Perugia, Italy; 2 LiLAIM, University for Foreigners of Perugia, Perugia, Italy

No further comments.

Is the work clearly and accurately presented and does it cite the current literature?

Yes

If applicable, is the statistical analysis and its interpretation appropriate?

Yes

Are all the source data underlying the results available to ensure full reproducibility?

Yes

Is the study design appropriate and is the work technically sound?

Yes

Are the conclusions drawn adequately supported by the results?

Yes

Are sufficient details of methods and analysis provided to allow replication by others?

Yes

Reviewer Expertise:

Artificial intelligence in language learning, online courses, educational technologies.

We confirm that we have read this submission and believe that we have an appropriate level of expertise to confirm that it is of an acceptable scientific standard.

# Reviewer report: Reviewer response for version 1

**DOI:** 10.5256/f1000research.181962.r437903

**Published:** 2026-01-06

**Authors:** William J Marin-Rodriguez

**Affiliations:** 1 Universidad Nacional José Faustino Sánchez Carrión, Huacho, Peru

**1. Summary of the article**

 The manuscript investigates how machine learning (ML) algorithms can be used to predict university teachers’ digital competence levels. Digital competence is assessed with the DigCompEdu Check-In questionnaire, adapted to Spanish, which yields proficiency levels from A1 (Newcomer) to C2 (Pioneer) across several domains (professional engagement, digital resources, pedagogy, assessment and feedback, and learner empowerment).

 Using data from faculty at two Ecuadorian universities, the authors first analyse associations between academic level and specific digital-competence items via chi-square tests. They then build and compare nine supervised ML models—Logistic Regression, Decision Tree, Random Forest, Gradient Boosting, k-Nearest Neighbours, Support Vector Machine, Stochastic Gradient Descent, Neural Network and Naïve Bayes—implemented in Orange. Model performance is evaluated mainly through AUC, classification accuracy, F1-score, precision, recall and Matthews correlation coefficient.

 The main findings are that (i) higher academic degrees are associated with higher levels of digital competence, and (ii) ensemble methods (Random Forest, Gradient Boosting) and Neural Networks outperform simpler models, particularly when classifying intermediate and advanced competence levels (B2, C1). Models perform less well at the lowest levels (A1, A2). The authors conclude that ML can support diagnostic assessment of faculty digital competence and inform targeted professional development initiatives.

**2. Major comments**

**2.1 Study design and technical soundness**

*(Question: “Is the study design appropriate and is the work technically sound?” – answered **Partly**)*

 Overall, the quantitative, cross-sectional design and the use of supervised ML to predict DigCompEdu-based competence levels are appropriate and align with the stated research questions. The focus on two institutions in Ecuador is clearly defined and relevant.

 However, several aspects of the technical implementation require clarification to fully support the claim that the study is technically sound:

**Sampling strategy and context**

 Please describe more precisely the *population* of interest (total number of faculty in each institution), the recruitment procedure (census vs. voluntary participation), inclusion/exclusion criteria, and the response rate.

 This information is essential to judge potential sampling biases and the representativeness of the dataset.

**Class imbalance and competence levels**

 Table 3 shows a highly imbalanced distribution across competence levels (e.g., 60 cases at A1 vs. 2,048 at B2 and 1,320 at C1).

 The manuscript should explicitly acknowledge this imbalance in the Methods section and explain whether any strategies were used to mitigate it (e.g., class weights, over/under-sampling). If no such strategies were applied, this should be clearly stated and discussed as a limitation, especially when interpreting poor performance at lower levels.

**Rationale for the choice of models**

 The selection of nine supervised algorithms is reasonable, but the rationale could be more explicit. A short paragraph contrasting “traditional” models (e.g., Logistic Regression) with ensemble and neural models, and explaining why these particular algorithms were chosen, would strengthen the design justification.

 You might also briefly compare the chosen classification framework with alternative approaches (e.g., ordinal regression or multi-level models) and explain why the chosen approach is preferable for your research aims.

**Must be addressed:** clearer description of the sampling strategy and dataset context, explicit discussion of class imbalance and how (or whether) it was handled, and a slightly stronger justification of the modelling choices.

**2.2 Methods transparency and reproducibility**

*(Questions: “Are sufficient details of methods and analysis provided to allow replication by others?” – **Partly**;*

*“Are all the source data underlying the results available?” – **Yes**)*

 It is a strong point of the article that the underlying dataset is shared openly and that the software environment (Orange 3.38.1) is specified. This clearly supports data availability.

 However, the current description of the ML workflow is too high-level to allow other researchers to reproduce the exact analyses and results:

**Definition of the target variable(s)**

 Please explain in more detail how the DigCompEdu scores were aggregated into the outcome used for prediction.

 Did you use a global competence level, domain-specific levels, or both?

 Where levels with very few participants (e.g., C2/Pioneer) merged into adjacent categories or excluded? If so, please describe the recoding explicitly.

**Pre-processing steps**

 Provide a concise but precise description of data pre-processing:

 Handling of missing responses (imputation method, listwise deletion, thresholds, etc.).

 Encoding socio-educational predictors (e.g., age bands, academic degree, years of experience, institution).

 Any normalization or scaling applied, particularly relevant for kNN, SVM, SGD and Neural Networks.

 Whether any feature selection or dimensionality-reduction steps were used before modelling.

**Validation protocol**

 In Orange’s Test & Score widget several options are available (n-fold cross-validation, hold-out, bootstrap, etc.). Please specify:

 Which validation strategy was used (e.g., 10-fold stratified cross-validation).

 The number of folds and whether the splitting was stratified by competence level.

 Whether results were averaged over multiple runs with different random seeds.

**Model configuration and hyperparameters**

 For each algorithm, please report the key hyperparameters and how they were chosen (default values vs. tuning):

 Decision Tree / Random Forest / Gradient Boosting: number of trees, maximum depth, splitting criterion, learning rate, etc.

 kNN: number of neighbours, distance metric, weighting scheme.

 SVM: kernel type, C and gamma parameters.

 SGD: loss function, regularisation, learning rate schedule.

 Neural Network: network architecture (number of layers and neurons), activation functions, optimisation algorithm, number of epochs, batch size, early stopping criteria.

 Naïve Bayes and Logistic Regression: any regularisation choices and class-weight settings.

 If all models used default settings in Orange, please state this explicitly; even default configurations should be reported so that others can replicate them.

**Releasing the analysis workflow**

 To further enhance reproducibility, I strongly encourage you to share the orange workflow file (.ows) in the repository alongside the dataset and provide the link in the Data availability section.

**Must be addressed:** explicit description of outcome construction, pre-processing decisions, validation strategy and model hyperparameters; ideally sharing the orange workflow file.

**2.3 Statistical analysis and interpretation**

*(Question: “If applicable, is the statistical analysis and its interpretation appropriate?” – **Partly**)*

 The use of chi-square tests to examine associations between academic level and individual competence items (Table 1) is appropriate and well-motivated. The choice of AUC, accuracy, F1-score, precision, recall and MCC as performance metrics is also suitable.

 Still, several improvements would make the statistical analysis more rigorous:

**Multiple comparisons and effect sizes**

 Because many chi-square tests are conducted, please specify the significance level used (e.g., α = 0.05) and discuss how multiple testing was handled.

 Even if you decide not to formally correct for multiple comparisons, acknowledging the issue and justifying your decision (e.g., exploratory rather than confirmatory analysis) would be valuable.

 I also recommend reporting an effect size measure such as Cramer’s V for each chi-square test. This would help readers gauge the magnitude, not just the existence, of the associations.

**Uncertainty in model performance**

 Model metrics are reported as point estimates only. Please provide a measure of uncertainty, such as standard deviation or 95% confidence intervals across cross-validation folds.

 This is particularly important when comparing algorithms whose AUC or F1 values differ only slightly.

**Model calibration and practical impact**

 If probability outputs are used or envisaged for decision-making (e.g., identifying teachers who are likely to be at low competence levels), a brief comment on model calibration would be useful. If calibration was not assessed, this can be mentioned as a limitation.

**Must be addressed:** explicit discussion of multiple testing and inclusion of effect sizes for chi-square analyses; some indication of uncertainty for model performance metrics.

**2.4 Conclusions and support from results**

*(Question: “Are the conclusions drawn adequately supported by the results?” – **Partly**)*

 In general, the key conclusions—higher academic degree associated with higher digital competence, and better predictive performance of ensemble and neural models at intermediate/advanced levels—are consistent with the reported empirical results.

 However, a few aspects could be refined to more faithfully reflect the evidence:

**Scope of generalisation**

 The data come from two universities in Ecuador. Statements that generalize findings to “Latin American higher education” or to university faculty in general should be toned down or more clearly framed as hypotheses for future research.

 Emphasizing that the results are context-specific will also help readers interpret the practical implications appropriately.

**Explicit mapping from hypotheses to results**

 The introduction formulates five hypotheses (H1–H5). In the Discussion or Conclusions, it would be helpful to explicitly revisit each hypothesis and state whether it was fully supported, partially supported or not supported, citing specific tables/figures.

 For instance, the difficulty in correctly identifying A1/A2 levels connects directly with H5 and deserves a clearer statement.

**Limitations and use in practice**

 The limitations section rightly mentions class imbalance and the absence of certain contextual variables. These limitations should also be more clearly echoed when recommending the use of the models for institutional decision-making, especially regarding the limited reliability at low competence levels.

**Must be addressed:** moderating claims that go beyond the empirical context; a clearer, hypothesis-by-hypothesis discussion of how the results support (or fail to support) H1–H5; and a stronger linkage between identified limitations and the practical recommendations.

**3. Presentation, language and minor issues**

*(Question: “Is the work clearly and accurately presented, and does it cite the current literature?” – **Yes**, with minor suggestions)*

 The manuscript is generally clear, well-structured and supported by a rich, up-to-date literature base on digital competence and ML in education. Figures and tables are informative.

 I offer a few minor suggestions to further improve clarity:

**Language and style**

 A careful language edit would help breaking up some very long sentences, ensuring subject–verb agreement, and correcting minor typographical issues (for example, removing the inverted Spanish question mark in the English research question).

 Check that all abbreviations (AUC, MCC, etc.) are defined at first mention in the main text or table captions.

**Competence levels**

 Please state explicitly whether any participants reached level C2 (Pioneer). If this level is absent from the analyses (e.g., because there were no cases or they were merged), this should be clearly explained.

**Sample size consistency (important)**

 The totals in the confusion matrices (Table 3) sum to 5,148 cases, whereas other parts of the article and materials mention 4,154 responses. This discrepancy must be resolved:

 If some records were excluded from certain analyses, or if multiple rows per participant were generated during preprocessing, please explain this in the Methods section and ensure that the same N is used consistently throughout the manuscript.

 Consistency in sample size reporting is crucial for transparency and interpretability.

**Must be addressed:** clarification and harmonisation of the reported sample size; explanation of the treatment of level C2. Other language and style issues are minor but recommended.

**4. Overall assessment**

 This manuscript addresses an important and timely topic at the intersection of digital pedagogy and machine learning. The combination of a large dataset, a recognised competence framework (DigCompEdu) and a broad set of ML models has the potential to make a useful contribution to literature and to institutional practice.

 However, to fully meet the F1000Research criteria of scientific soundness and reproducibility, I believe the authors should address the points highlighted above—most notably: (1) clarifying the sampling strategy and reconciling sample sizes, (2) providing a much more detailed description of the ML pipeline (pre-processing, validation, hyperparameters), and (3) strengthening the statistical reporting and the alignment between hypotheses and results.

 Once these issues are resolved, I would consider the paper to be scientifically robust and suitable for indexing. At its current stage, my overall recommendation would be **Approved with reservations**.

**1. Summary of the article**

 The manuscript investigates how machine learning (ML) algorithms can be used to predict university teachers’ digital competence levels. Digital competence is assessed with the DigCompEdu Check-In questionnaire, adapted to Spanish, which yields proficiency levels from A1 (Newcomer) to C2 (Pioneer) across several domains (professional engagement, digital resources, pedagogy, assessment and feedback, and learner empowerment).

 Using data from faculty at two Ecuadorian universities, the authors first analyse associations between academic level and specific digital-competence items via chi-square tests. They then build and compare nine supervised ML models—Logistic Regression, Decision Tree, Random Forest, Gradient Boosting, k-Nearest Neighbours, Support Vector Machine, Stochastic Gradient Descent, Neural Network and Naïve Bayes—implemented in Orange. Model performance is evaluated mainly through AUC, classification accuracy, F1-score, precision, recall and Matthews correlation coefficient.

 The main findings are that (i) higher academic degrees are associated with higher levels of digital competence, and (ii) ensemble methods (Random Forest, Gradient Boosting) and Neural Networks outperform simpler models, particularly when classifying intermediate and advanced competence levels (B2, C1). Models perform less well at the lowest levels (A1, A2). The authors conclude that ML can support diagnostic assessment of faculty digital competence and inform targeted professional development initiatives.

**2. Major comments**

**2.1 Study design and technical soundness**

*(Question: “Is the study design appropriate and is the work technically sound?” – answered **Partly**)*

 Overall, the quantitative, cross-sectional design and the use of supervised ML to predict DigCompEdu-based competence levels are appropriate and align with the stated research questions. The focus on two institutions in Ecuador is clearly defined and relevant.

 However, several aspects of the technical implementation require clarification to fully support the claim that the study is technically sound:

**Sampling strategy and context**

 Please describe more precisely the *population* of interest (total number of faculty in each institution), the recruitment procedure (census vs. voluntary participation), inclusion/exclusion criteria, and the response rate.

 This information is essential to judge potential sampling biases and the representativeness of the dataset.

**Class imbalance and competence levels**

 Table 3 shows a highly imbalanced distribution across competence levels (e.g., 60 cases at A1 vs. 2,048 at B2 and 1,320 at C1).

 The manuscript should explicitly acknowledge this imbalance in the Methods section and explain whether any strategies were used to mitigate it (e.g., class weights, over/under-sampling). If no such strategies were applied, this should be clearly stated and discussed as a limitation, especially when interpreting poor performance at lower levels.

**Rationale for the choice of models**

 The selection of nine supervised algorithms is reasonable, but the rationale could be more explicit. A short paragraph contrasting “traditional” models (e.g., Logistic Regression) with ensemble and neural models, and explaining why these particular algorithms were chosen, would strengthen the design justification.

 You might also briefly compare the chosen classification framework with alternative approaches (e.g., ordinal regression or multi-level models) and explain why the chosen approach is preferable for your research aims.

**Must be addressed:** clearer description of the sampling strategy and dataset context, explicit discussion of class imbalance and how (or whether) it was handled, and a slightly stronger justification of the modelling choices.

**2.2 Methods transparency and reproducibility**

*(Questions: “Are sufficient details of methods and analysis provided to allow replication by others?” – **Partly**;*

*“Are all the source data underlying the results available?” – **Yes**)*

 It is a strong point of the article that the underlying dataset is shared openly and that the software environment (Orange 3.38.1) is specified. This clearly supports data availability.

 However, the current description of the ML workflow is too high-level to allow other researchers to reproduce the exact analyses and results:

**Definition of the target variable(s)**

 Please explain in more detail how the DigCompEdu scores were aggregated into the outcome used for prediction.

 Did you use a global competence level, domain-specific levels, or both?

 Where levels with very few participants (e.g., C2/Pioneer) merged into adjacent categories or excluded? If so, please describe the recoding explicitly.

**Pre-processing steps**

 Provide a concise but precise description of data pre-processing:

 Handling of missing responses (imputation method, listwise deletion, thresholds, etc.).

 Encoding socio-educational predictors (e.g., age bands, academic degree, years of experience, institution).

 Any normalization or scaling applied, particularly relevant for kNN, SVM, SGD and Neural Networks.

 Whether any feature selection or dimensionality-reduction steps were used before modelling.

**Validation protocol**

 In Orange’s Test & Score widget several options are available (n-fold cross-validation, hold-out, bootstrap, etc.). Please specify:

 Which validation strategy was used (e.g., 10-fold stratified cross-validation).

 The number of folds and whether the splitting was stratified by competence level.

 Whether results were averaged over multiple runs with different random seeds.

**Model configuration and hyperparameters**

 For each algorithm, please report the key hyperparameters and how they were chosen (default values vs. tuning):

 Decision Tree / Random Forest / Gradient Boosting: number of trees, maximum depth, splitting criterion, learning rate, etc.

 kNN: number of neighbours, distance metric, weighting scheme.

 SVM: kernel type, C and gamma parameters.

 SGD: loss function, regularisation, learning rate schedule.

 Neural Network: network architecture (number of layers and neurons), activation functions, optimisation algorithm, number of epochs, batch size, early stopping criteria.

 Naïve Bayes and Logistic Regression: any regularisation choices and class-weight settings.

 If all models used default settings in Orange, please state this explicitly; even default configurations should be reported so that others can replicate them.

**Releasing the analysis workflow**

 To further enhance reproducibility, I strongly encourage you to share the orange workflow file (.ows) in the repository alongside the dataset and provide the link in the Data availability section.

**Must be addressed:** explicit description of outcome construction, pre-processing decisions, validation strategy and model hyperparameters; ideally sharing the orange workflow file.

**2.3 Statistical analysis and interpretation**

*(Question: “If applicable, is the statistical analysis and its interpretation appropriate?” – **Partly**)*

 The use of chi-square tests to examine associations between academic level and individual competence items (Table 1) is appropriate and well-motivated. The choice of AUC, accuracy, F1-score, precision, recall and MCC as performance metrics is also suitable.

 Still, several improvements would make the statistical analysis more rigorous:

**Multiple comparisons and effect sizes**

 Because many chi-square tests are conducted, please specify the significance level used (e.g., α = 0.05) and discuss how multiple testing was handled.

 Even if you decide not to formally correct for multiple comparisons, acknowledging the issue and justifying your decision (e.g., exploratory rather than confirmatory analysis) would be valuable.

 I also recommend reporting an effect size measure such as Cramer’s V for each chi-square test. This would help readers gauge the magnitude, not just the existence, of the associations.

**Uncertainty in model performance**

 Model metrics are reported as point estimates only. Please provide a measure of uncertainty, such as standard deviation or 95% confidence intervals across cross-validation folds.

 This is particularly important when comparing algorithms whose AUC or F1 values differ only slightly.

**Model calibration and practical impact**

 If probability outputs are used or envisaged for decision-making (e.g., identifying teachers who are likely to be at low competence levels), a brief comment on model calibration would be useful. If calibration was not assessed, this can be mentioned as a limitation.

**Must be addressed:** explicit discussion of multiple testing and inclusion of effect sizes for chi-square analyses; some indication of uncertainty for model performance metrics.

**2.4 Conclusions and support from results**

*(Question: “Are the conclusions drawn adequately supported by the results?” – **Partly**)*

 In general, the key conclusions—higher academic degree associated with higher digital competence, and better predictive performance of ensemble and neural models at intermediate/advanced levels—are consistent with the reported empirical results.

 However, a few aspects could be refined to more faithfully reflect the evidence:

**Scope of generalisation**

 The data come from two universities in Ecuador. Statements that generalize findings to “Latin American higher education” or to university faculty in general should be toned down or more clearly framed as hypotheses for future research.

 Emphasizing that the results are context-specific will also help readers interpret the practical implications appropriately.

**Explicit mapping from hypotheses to results**

 The introduction formulates five hypotheses (H1–H5). In the Discussion or Conclusions, it would be helpful to explicitly revisit each hypothesis and state whether it was fully supported, partially supported or not supported, citing specific tables/figures.

 For instance, the difficulty in correctly identifying A1/A2 levels connects directly with H5 and deserves a clearer statement.

**Limitations and use in practice**

 The limitations section rightly mentions class imbalance and the absence of certain contextual variables. These limitations should also be more clearly echoed when recommending the use of the models for institutional decision-making, especially regarding the limited reliability at low competence levels.

**Must be addressed:** moderating claims that go beyond the empirical context; a clearer, hypothesis-by-hypothesis discussion of how the results support (or fail to support) H1–H5; and a stronger linkage between identified limitations and the practical recommendations.

**3. Presentation, language and minor issues**

*(Question: “Is the work clearly and accurately presented, and does it cite the current literature?” – **Yes**, with minor suggestions)*

 The manuscript is generally clear, well-structured and supported by a rich, up-to-date literature base on digital competence and ML in education. Figures and tables are informative.

 I offer a few minor suggestions to further improve clarity:

**Language and style**

 A careful language edit would help breaking up some very long sentences, ensuring subject–verb agreement, and correcting minor typographical issues (for example, removing the inverted Spanish question mark in the English research question).

 Check that all abbreviations (AUC, MCC, etc.) are defined at first mention in the main text or table captions.

**Competence levels**

 Please state explicitly whether any participants reached level C2 (Pioneer). If this level is absent from the analyses (e.g., because there were no cases or they were merged), this should be clearly explained.

**Sample size consistency (important)**

 The totals in the confusion matrices (Table 3) sum to 5,148 cases, whereas other parts of the article and materials mention 4,154 responses. This discrepancy must be resolved:

 If some records were excluded from certain analyses, or if multiple rows per participant were generated during preprocessing, please explain this in the Methods section and ensure that the same N is used consistently throughout the manuscript.

 Consistency in sample size reporting is crucial for transparency and interpretability.

**Must be addressed:** clarification and harmonisation of the reported sample size; explanation of the treatment of level C2. Other language and style issues are minor but recommended.

**4. Overall assessment**

 This manuscript addresses an important and timely topic at the intersection of digital pedagogy and machine learning. The combination of a large dataset, a recognised competence framework (DigCompEdu) and a broad set of ML models has the potential to make a useful contribution to literature and to institutional practice.

 However, to fully meet the F1000Research criteria of scientific soundness and reproducibility, I believe the authors should address the points highlighted above—most notably: (1) clarifying the sampling strategy and reconciling sample sizes, (2) providing a much more detailed description of the ML pipeline (pre-processing, validation, hyperparameters), and (3) strengthening the statistical reporting and the alignment between hypotheses and results.

Is the work clearly and accurately presented and does it cite the current literature?

Yes

If applicable, is the statistical analysis and its interpretation appropriate?

Partly

Are all the source data underlying the results available to ensure full reproducibility?

Yes

Is the study design appropriate and is the work technically sound?

Partly

Are the conclusions drawn adequately supported by the results?

Partly

Are sufficient details of methods and analysis provided to allow replication by others?

Partly

Reviewer Expertise:

Data science

I confirm that I have read this submission and believe that I have an appropriate level of expertise to confirm that it is of an acceptable scientific standard, however I have significant reservations, as outlined above.

# Response

**Published:** 2026-01-21

**Authors:** JENNIFFER SOBEIDA MOREIRA CHOEZ

Response to Reviewer 

https://drive.google.com/drive/folders/1j2a6RB7DTboqr2u65CIsBbIk1ivZYTGc?usp=sharing

**1) Sampling strategy and dataset context (population, recruitment, inclusion/exclusion, response rate)**

 The Methods section incorporated a more precise characterization of the population of interest and the recruitment strategy. Specifically, the reference population was reported for each institution and in total, and participation was described as a voluntary-response process based on institutional dissemination rather than a census. In addition, the analytic sample size and the response rate were reported explicitly. Finally, eligibility rules were operationalized through explicit inclusion/exclusion criteria presented in a dedicated table to document how the analytic dataset was delimited and quality-controlled.

**2) Definition of the target variable (DigCompEdu outcome construction and category handling)**

 The Methods section clarified the construction of the prediction target by stating that item-level responses were organized in long format and that the modelling target corresponded to a single global DigCompEdu proficiency label. Moreover, the final outcome space was documented as comprising the observed categories A1–C1, and it was stated that no category fusion/recoding was required because upper sparse levels (e.g., C2) did not appear in the analytic dataset.

**3) Preprocessing steps (missing data, encoding, scaling, feature selection)**

 The Methods section included a concise but explicit preprocessing description. Data integrity controls were documented (deduplication and quality screening), missingness screening was reported with the resulting decision not to impute, predictors were specified as categorical and encoded via one-hot indicators, scaling was reported as unnecessary under the 0/1 indicator representation, and feature selection/dimensionality reduction was stated as not implemented because the predictor set remained parsimonious and theory-aligned.

**4) Class imbalance (explicit acknowledgment and handling strategy)**

 The Methods section explicitly acknowledged the imbalance in the target distribution across proficiency levels and stated that no rebalancing strategy (class weights, over-/under-sampling) was applied; consequently, models were trained under the empirically observed proportions and evaluation emphasized metrics informative under imbalance and class-wise inspection through confusion matrices. In addition, the Conclusions/limitations text reinforced the practical implication of reduced sensitivity for low-frequency strata.

**5) Validation protocol and hyperparameters (replicability of Orange workflow)**

 The Methods section specified that performance estimation relied on stratified k-fold cross-validation within the Orange “Test and Score” widget, and it clarified that results were computed in a single evaluation run under default randomization settings (i.e., not averaged over multiple random seeds). Furthermore, it was stated that models were trained using default Orange widget configurations and that no hyperparameter optimization was executed; the manuscript also indicated that key learner hyperparameters were documented in a dedicated table to support replication.

**6) Multiple comparisons and effect sizes for chi-square analyses**

 The Methods section clarified inferential settings by stating the nominal two-sided alpha threshold and by addressing multiplicity through false discovery rate control via the Benjamini–Hochberg adjustment, with decisions based on adjusted p-values. In addition, Cramer’s V was reported as the effect-size measure to support interpretation of practical relevance beyond statistical significance and to improve comparability across items.

**7) Uncertainty in model performance (dispersion and confidence intervals)**

 Model performance reporting was expanded beyond point estimates by summarizing fold-wise values as mean and standard deviation across folds for each metric and by computing 95% confidence intervals for the cross-validated mean using a t-based approach. Consequently, algorithm comparisons accounted for both central tendency and dispersion, and small differences were interpreted conservatively under overlapping uncertainty intervals.

**8) Model calibration and practical impact (probabilities, limitations, and deployment implications)**

 The manuscript clarified that predicted probabilities were treated as auxiliary outputs rather than operational thresholds and stated that calibration was not formally evaluated in the current workflow. Therefore, probability values were interpreted as relative indicators of model confidence, and prospective institutional use based on probability cut-offs was framed as requiring explicit calibration assessment and threshold selection aligned with institutional priorities, error costs, and prevalence of low-proficiency categories. This limitation was reiterated in the concluding recommendations as a condition for future deployment-oriented work.

**9) Justification of model choice and comparison with alternative frameworks (ordinal/multilevel)**

 Response. The Methods section strengthened the modelling rationale by contrasting transparent baseline classifiers with higher-capacity ensemble and neural approaches and by explaining why this panel enabled principled comparison of performance trade-offs under a unified protocol. Moreover, the outcome formulation as multiclass classification was justified in relation to the diagnostic nature of DigCompEdu proficiency categories; ordinal regression and multilevel modelling were acknowledged as alternatives, but the manuscript explained why they were not preferred for the present predictive-discrimination objective given their stronger structural assumptions and different inferential focus.

**10) Conclusions: scope of generalisation, hypothesis-to-results mapping (H1–H5), and linkage to limitations/practice**

 The Conclusions were refined to better align claims with the empirical scope by emphasizing transferability constraints tied to context and missing contextual variables, and by framing broader applicability as a matter for external validation. In addition, the Conclusions incorporated an explicit hypothesis-by-hypothesis statement that reported whether each hypothesis was supported or partially supported, including a direct linkage between weaker identification of low-proficiency strata and the hypothesis focused on prediction difficulty. Finally, limitations were explicitly connected to practical recommendations by noting that imbalance and the absence of rebalancing constrained sensitivity for minority strata and that institutional use should prioritize lower-stakes, group-level planning unless further calibration/imbalance-handling steps were implemented.

# Response

**Published:** 2026-07-17

**Authors:** JENNIFFER SOBEIDA MOREIRA CHOEZ

We sincerely thank the reviewer for the thorough, constructive, and insightful evaluation of our manuscript. We greatly appreciate the time devoted to assessing our work and the valuable recommendations provided. We are pleased that the reviewer recognizes the relevance of the research topic, the appropriateness of the quantitative design, the use of the DigCompEdu framework, the openness of the dataset, and the potential contribution of the study to the field of digital education and machine learning.

 We respectfully note that many of the issues raised have already been incorporated into the revised version of the manuscript following the recommendations of the previous reviewers. Throughout the revision process, particular attention was devoted to improving methodological transparency, reproducibility, statistical reporting, and the alignment between the research hypotheses and the empirical findings. Consequently, we believe that the current version fully satisfies the methodological and reporting standards expected for studies involving supervised machine learning in educational research while preserving the original scientific contribution of the work.

 Below we respond point-by-point.

**Comment 2.1. Study design and technical robustness**

 Regarding the sampling strategy, we respectfully indicate that the revised manuscript already provides a substantially expanded description of the target population, institutional context, recruitment strategy, eligibility criteria, inclusion and exclusion criteria, voluntary participation process, and response rate. These additions were incorporated precisely to strengthen methodological transparency and enable readers to properly assess the representativeness and scope of the study.

 Concerning class imbalance, we agree that imbalance is an inherent characteristic of educational datasets based on naturally occurring competence distributions. Rather than artificially modifying the empirical distribution through oversampling or undersampling procedures, we deliberately preserved the observed class frequencies to maintain ecological validity and avoid introducing synthetic patterns that could compromise the interpretability of institutional diagnostic models. This decision follows current recommendations in educational machine learning, where preserving naturally occurring distributions is considered appropriate when the objective is predictive assessment rather than algorithmic optimization. Accordingly, the manuscript explicitly recognizes this imbalance as a methodological limitation and discusses its implications for the lower predictive performance observed in minority competence levels.

 Regarding the selection of machine learning algorithms, we respectfully note that the revised manuscript now provides a comprehensive theoretical justification explaining the inclusion of traditional statistical learners, ensemble methods, margin-based classifiers, instance-based learners, and neural networks. The purpose was not to identify a universally superior algorithm but rather to establish a robust comparative framework that evaluates different inductive biases under identical experimental conditions. We also clarify why multiclass classification was preferred over ordinal regression or multilevel modeling, since the primary objective of this study was predictive discrimination across competence categories rather than causal inference or hierarchical variance decomposition. These methodological clarifications strengthen the scientific rationale underlying the modeling strategy without modifying the analytical design.

**Comment 2.2. Transparency and reproducibility**

 We respectfully indicate that the revised manuscript substantially expands the methodological description in order to facilitate reproducibility. Specifically, the manuscript now provides:a detailed explanation of the construction of the target variable; explicit description of the long-format data structure; preprocessing procedures; handling of missing data; predictor encoding; feature engineering decisions; validation strategy; hyperparameter specification; justification for using Orange Data Mining; and the complete machine learning workflow.

 These additions were incorporated precisely to address reproducibility requirements and to ensure that future researchers can replicate the analytical process with a high degree of fidelity. Importantly, all algorithms were intentionally evaluated under identical default configurations to guarantee a fair methodological comparison across models rather than optimizing individual algorithms independently, which was consistent with the comparative objective of the study.

 Regarding the recommendation to share the Orange workflow (.ows), we appreciate this valuable suggestion. Although the manuscript already provides sufficient methodological detail for replication, we recognize that including the workflow file as supplementary material would further strengthen computational reproducibility. We therefore consider this recommendation an excellent opportunity to improve transparency in future updates of the repository.

**Comment 2.3. Statistical analysis**

 The revised manuscript already incorporates several of these recommendations. Specifically, statistical inference now explicitly reports the nominal significance level and addresses the issue of multiple hypothesis testing using the Benjamini-Hochberg False Discovery Rate correction, which is widely recommended for exploratory analyses involving multiple comparisons. Furthermore, effect sizes based on Cramer's V were incorporated to complement statistical significance with practical relevance, allowing readers to interpret not only whether associations exist but also their magnitude.

 Similarly, model evaluation was strengthened by reporting fold-based uncertainty measures derived from cross-validation, including variability across folds and confidence intervals for performance metrics. Consequently, algorithm comparisons are no longer interpreted solely on the basis of point estimates but rather considering their associated uncertainty, thereby providing a more rigorous statistical interpretation.

 Regarding calibration, we agree that probability calibration represents an important aspect for future implementation in decision-support systems. Nevertheless, the principal objective of the present study was comparative evaluation of predictive discrimination rather than deployment of calibrated probabilistic decision models. For this reason, calibration was appropriately acknowledged as a future research direction rather than as a methodological deficiency of the current work.

**Comment 2.4. Conclusions and interpretation**

 Following recommendations received during previous rounds of peer review, the Discussion and Conclusions sections were substantially revised to ensure a closer correspondence between the reported evidence and the interpretations presented. The revised manuscript now explicitly limits the generalizability of the findings to the institutional context investigated and avoids extending conclusions beyond the empirical scope of the study.

 Likewise, the discussion now explicitly addresses each research hypothesis, indicating whether it was fully supported, partially supported, or not supported by the empirical evidence. This hypothesis-by-hypothesis interpretation strengthens the logical consistency between the research objectives, statistical analyses, and final conclusions.

 Finally, the limitations section was expanded to discuss the implications of class imbalance and contextual variables for institutional implementation, emphasizing that predictive models should complement rather than replace educational decision-making processes. These revisions ensure that practical recommendations remain scientifically justified and proportionate to the available evidence.

**Comment 3. Presentation and language**

 Following previous reviewers' recommendations, the manuscript underwent comprehensive language editing to improve readability, consistency, and scientific precision. Typographical issues, abbreviation definitions, terminology consistency, and stylistic refinements were carefully revised throughout the manuscript.

 Regarding the absence of the C2 (Pioneer) competence level, the revised manuscript explicitly clarifies that no participants reached this category; consequently, no category merging or recoding procedures were required.

 Similarly, the apparent discrepancy between the respondent sample (234 participants) and the modeling dataset (5,148 observations) has been clarified in the Methods section. Specifically, the machine learning analysis was performed using an item-level long-format dataset derived from the 22 DigCompEdu items answered by each participant (234 × 22 = 5,148 observations). Therefore, these values represent different analytical units rather than inconsistent sample sizes, fully preserving methodological coherence and reproducibility.

 We sincerely thank the reviewer once again for the thoughtful evaluation and valuable recommendations. The comments have contributed to further strengthening the methodological transparency and scientific rigor of the manuscript.

 At the same time, we respectfully emphasize that the current revised version already incorporates the vast majority of the suggested improvements as a result of the previous peer-review process. These revisions substantially enhanced the description of the sampling strategy, machine learning workflow, statistical analyses, reproducibility, hypothesis evaluation, limitations, and reporting consistency, while preserving the original analytical design and scientific contribution of the study.

 We therefore believe that the manuscript now provides a robust, transparent, and reproducible methodological framework, fully supported by the empirical evidence presented, and that it satisfies the scientific standards expected for publication in *F1000Research*.

# Reviewer report: Reviewer response for version 1

**DOI:** 10.5256/f1000research.181962.r391202

**Published:** 2025-07-28

**Authors:** Giorgia Montanucci, Talia Sbardella

**Affiliations:** 1 University for Foreigners of Perugia, Perugia, Italy; 2 LiLAIM, University for Foreigners of Perugia, Perugia, Italy

This manuscript is a valuable interdisciplinary contribution, bridging artificial intelligence, teacher training, and digital pedagogy. The authors present a clear and well-executed study that explores the predictive potential of nine supervised machine learning algorithms using a large dataset of faculty responses (n = 4,154) collected from two Ecuadorian institutions. Their central claim, that ensemble methods and neural networks outperform simpler models in identifying advanced levels of digital competence, is supported by an appropriate methodological design and comprehensive performance metrics. The use of the DigCompEdu framework and the attention to ethical procedures, including informed consent and IRB approval, reinforce the quality of the study.

 Moreira-Choez and colleagues offer a well-scoped empirical investigation that highlights the value of advanced machine learning techniques in identifying digital competency patterns among university staff. While the study does not necessarily introduce new technical methods, it provides a strong application of existing tools within a specific educational context. It represents an important step forward, especially in regions where empirical evidence on faculty digital skills remains scarce. The inclusion of multiple models is a strength, as is the attempt to interpret the results using visual tools from the Orange platform.

 That said, some limitations warrant further reflection. In particular, the visual representation of data (especially Figures 2–4) is complex and could be overwhelming to non-specialist readers. Additionally, Figure 1 is difficult to read; improving its clarity would significantly enhance the reader’s comprehension of the presented data. Simplified visual summaries or key takeaways could help.

 It could also be valuable to extend the study to additional universities and/or educational contexts, in order to compare findings across institutions and enhance the external validity of the results. This would contribute to a broader understanding of how digital competencies manifest in diverse academic settings.

 One area that opens new possibilities for future work relates to feature interpretability. While the use of Orange’s Explain Model widget is mentioned, the paper stops short of identifying which features most strongly influenced the predictions across the different algorithms. Understanding whether variables such as age, academic degree, or teaching experience were the strongest predictors in high-performing models could inform more targeted faculty development strategies. This would be particularly relevant if the models are to be adopted in real-world institutional contexts.

 In summary, the study presents a solid and methodologically rigorous analysis that supports the use of machine learning for assessing digital competencies in higher education. The manuscript as a whole is well-conceived and clearly written.

Is the work clearly and accurately presented and does it cite the current literature?

Yes

If applicable, is the statistical analysis and its interpretation appropriate?

Yes

Are all the source data underlying the results available to ensure full reproducibility?

Yes

Is the study design appropriate and is the work technically sound?

Yes

Are the conclusions drawn adequately supported by the results?

Yes

Are sufficient details of methods and analysis provided to allow replication by others?

Yes

Reviewer Expertise:

Artificial intelligence in language learning, online courses, educational technologies.

We confirm that we have read this submission and believe that we have an appropriate level of expertise to confirm that it is of an acceptable scientific standard.

# Response

**Published:** 2026-01-21

**Authors:** JENNIFFER SOBEIDA MOREIRA CHOEZ

**Reviewer 1 – Response**

 We sincerely thank the reviewer for the thorough and constructive evaluation of our manuscript, as well as for recognizing its interdisciplinary contribution at the intersection of artificial intelligence, teacher training, and digital pedagogy. We greatly appreciate the positive assessment of the methodological rigor, ethical standards, and relevance of the study, particularly within underrepresented regional contexts.

 Below, we address the specific observations and suggestions raised.

https://drive.google.com/drive/folders/1j2a6RB7DTboqr2u65CIsBbIk1ivZYTGc?usp=drive_link

**1. Clarity and complexity of visual representations (Figures 1–4)**

**The reviewer noted that some visual representations, particularly Figures 2–4, may be complex for non-specialist readers, and that Figure 1 was difficult to read. Simplified summaries or clearer visual presentation were suggested.**

***Response.***

 We agree with this observation and have revised the manuscript accordingly. Figure 1 was redesigned to improve readability, including clearer labeling, improved spacing, and a simplified workflow representation. In addition, explanatory notes were added below the figure to guide interpretation.

 For Figures 2–4, supplementary clarifications were incorporated into the figure captions and the Results section to highlight key takeaways and reduce cognitive load for non-specialist readers. The narrative accompanying these figures was also streamlined to explicitly direct attention to the most relevant patterns, thereby improving accessibility without sacrificing analytical depth.

**2. Extension to additional universities and external validity**

**The reviewer suggested that extending the study to additional universities or educational contexts could strengthen external validity.**

***Response.***

 We fully concur with this recommendation. In the revised manuscript, we explicitly moderated claims of generalizability and clarified that the findings are context-specific to the two participating Ecuadorian universities. Moreover, the Conclusions section now frames multi-institutional and cross-context validation as a key direction for future research, emphasizing the importance of comparative studies across diverse higher education systems to enhance external validity.

**3. Feature interpretability and explanatory insights**

**The reviewer highlighted the potential value of deeper feature interpretability, particularly identifying which socio-educational variables most strongly influenced predictions in high-performing models.**

***Response.***

 We appreciate this insightful suggestion. In response, we expanded the methodological description of the Explain Model component in the Orange workflow and clarified its role in assessing feature contributions. While a full comparative ranking of predictors across all algorithms was beyond the scope of the current study, we explicitly acknowledged this limitation and emphasized feature-level interpretability as a critical avenue for future work.

 The Discussion and Conclusions sections now underline how identifying dominant predictors (e.g., academic degree, age, or teaching level) could directly inform targeted faculty development strategies and support responsible institutional deployment of predictive models.

 We are grateful for the reviewer’s overall positive evaluation of the study’s clarity, technical soundness, statistical validity, and reproducibility. No further revisions were requested regarding these aspects, and we are pleased that the reviewer found the conclusions to be adequately supported by the results.
